# How topic content shapes LLM personality-tailored persuasion: semantic anchoring and topic stereotype effects

**DOI:** 10.3389/fpsyt.2026.1756792

**Published:** 2026-01-30

**Authors:** Shuang Xu, Zili Zhou, Nan Zhao

**Affiliations:** 1 State Key Laboratory of Cognitive Science and Mental Health, Institute of Psychology, Chinese Academy of Sciences, Beijing, China; 2 Department of Psychology, University of Chinese Academy of Sciences, Beijing, China

**Keywords:** AI-generated messages, Big Five personality, large language models, personality-tailored persuasion, personalized communication, persuasive communication, semantic anchoring, topic stereotypes

## Abstract

Large language models (LLMs) have shown promise in generating personality-tailored persuasive messages, yet their effectiveness remains inconsistent across contexts. This research systematically investigated how the characteristics of recommended products or actions shapes the efficacy of LLM- generated personality-tailored persuasion through three experimental studies (N = 618). Study 1 revealed that personality-matching effects were limited and inconsistent when the core features of the recommended product or action were not controlled. Qualitative analysis suggested that uncontrolled semantic variation across personality framings obscured persuasive effects. Study 2 demonstrated that explicitly anchoring messages to core product or action features—while allowing stylistic variation—produced robust personality-matching effects across multiple traits and topics. Study 3 extended findings across diverse domains (health, consumer products, entertainment, prosocial behavior) and confirmed that topic-specific stereotypes systematically influence message effectiveness independent of recipient personality. Messages aligning with widely shared topic expectations (e.g., high-Extraversion framing for music festivals, high-Agreeableness framing for donations) were preferred across audiences regardless of individual traits. These findings reveal two critical boundary conditions for LLM-based personalized persuasion: stabilizing core content through semantic anchoring facilitates the emergence of personality-matching effects, and topic stereotypes create baseline preferences that may amplify or attenuate personalization benefits. Practically, effective implementation requires anchoring core content while modulating style, and evaluating topic stereotypes before applying trait customization. This work clarifies when and how generative AI can reliably enhance persuasive communication across mental health, public health, and consumer domains. Study 3 extended the findings across diverse domains (health, consumer products, entertainment, and prosocial behavior) and suggested a consistent role of topic-specific stereotypes in shaping message effectiveness, above and beyond recipient personality.

## 1 Introduction

Persuasive communication exerts a profound and pervasive influence on human life. Within mental health contexts, persuasive messages can motivate individuals to cultivate adaptive habits (e.g., maintaining regular mindfulness practice or attending therapy (1)), facilitate help-seeking (e.g., engaging in counseling or psychiatric care (2, 3)), and modify maladaptive cognitions that undermine psychological well-being (e.g., rumination or self-stigma (4, 5)). Comparable mechanisms underpin persuasive interventions across diverse societal domains, including public health (e.g., vaccination campaigns (6–8)), environmental sustainability (e.g., energy conservation (9, 10)), political participation (e.g., voter turnout (11, 12)), and consumer behavior (e.g., adoption of ethical products (13, 14)). Designing and delivering messages that consistently and effectively shape beliefs, attitudes, and behaviors across these contexts remains a central challenge for both researchers and practitioners.

Extensive research indicates that tailoring persuasive messages to the audience’s psychological characteristics enhances their effectiveness. Specifically, aligning message features with recipients’ enduring traits—such as personality, motivations, attitudes, or identity—can heighten perceived relevance and self-referencing, thereby facilitating attitude and behavioral change (15–17). This alignment is particularly consequential in mental health contexts, where generic interventions frequently suffer from low adherence and disengagement. Recent studies demonstrate that personalization strategies can substantially improve adherence to web-based intervention protocols (1) and foster sustained engagement with digital therapeutic resources (18, 19). Notably, personalization yields stronger effects when it targets psychologically meaningful dimensions rather than relying on superficial cues (20–22). The Big Five personality framework, the dominant taxonomy in personality psychology, has shown strong empirical robustness, cross-cultural replicability, and broad coverage of trait variation (23, 24). Specifically, prior research has shown that Big Five traits are systematically associated with individuals’ responses to persuasive messages (25–28).

The rapid emergence of large language models has opened unprecedented opportunities for automating persuasive message generation. Recent evidence suggests that LLM-produced texts can match or even surpass human-authored content in persuasive impact (29–35). This development raises an important question: Can LLMs be harnessed to generate personalized persuasive messages that account for individual differences, such as personality traits? Emerging empirical findings offer encouraging evidence. For example, Simchon et al. (36) demonstrated that ChatGPT-generated political advertisements congruent with recipients’ Openness were significantly more persuasive than incongruent versions. Extending these findings, Matz et al. (37) conducted four studies comprising seven experiments and found that GPT-generated personalized messages consistently outperformed generic baselines across consumer, political, and health domains. Personality-matched messages were perceived as more persuasive—particularly for Extraversion and Openness—and, in some cases, enhanced participants’ willingness to pay for target products.

Nonetheless, findings on the efficacy of LLM-based personalized persuasion remain mixed. Large-scale experiments have shown that dynamically targeted AI-generated messages do not always outperform non-targeted versions (38). Similarly, Xu and Zhao (39) found that personality-label-based prompting (e.g., “high openness”) elicited personality-matching effects only under limited topic conditions. Taken together, these results suggest that the success of LLM-driven personalization is not unconditional but shaped by identifiable boundary conditions. Given the expanding role of generative AI in shaping persuasive communication in diverse domains, clarifying when and how such personalization succeeds is theoretically and societally imperative (22).

Many factors shape persuasive effectiveness, and the inherent content requirements of the recommended product or action constitute a particularly consequential boundary condition. Persuasion research shows that argument quality—the strength, relevance, and evidentiary support of a message—critically determines effectiveness under conditions of thoughtful processing (40). Yet what counts as strong argumentation varies systematically across domains: health-behavior messages achieve maximal effectiveness when they successfully target perceived barriers, benefits, self-efficacy, and threat perceptions (41, 42); environmental persuasion proves most compelling when messages activate biospheric values and affirm prosocial self-concepts (43); whereas consumer persuasion is strengthened when content aligns with the brand’s dominant concept—whether functional, symbolic, or experiential (44). These domain-specific patterns indicate that each recommended product or action imposes distinct semantic and functional requirements —or “core features,” such as the necessity of highlighting protective efficacy for a vaccine or practical utility for a smartwatch—that constrain which message content can produce persuasive outcomes; thus, even personality-tailored messages should also satisfy these specific content demands to achieve meaningful impact. This observation suggests a potential solution: anchoring the key semantic features of the recommended product or action in the prompt may help stabilize personality-tailored messages while preserving topic-central content. We refer to this prompt-design principle as semantic anchoring.

Concurrently, audiences may hold preexisting stereotyped perceptions of different products or actions. Research on brand and product personality demonstrates that consumers reliably attribute human-like traits to brands and products, forming stable and measurable personality impressions (45–47). Accordingly, some topic-linked impressions can be meaningfully characterized in personality terms and may partially overlap with the Big Five framework (48). Importantly, such topic stereotypes can shape the effectiveness of personality-tailored persuasion by predisposing audiences to experience certain personality framings as more natural, appropriate, or credible than others. For example, health-prevention topics may be perceived as “responsible” or “careful,” whereas high-tech product may be viewed as “curious” or “innovative.” These implicit stereotypes may systematically advantage some personality framings of message over others, thereby obscuring or amplifying apparent matching.

Despite growing evidence that perceptions of the product or action critically shape persuasive outcomes, research on LLM-based personality-tailored persuasion has paid limited attention to this factor. To address this gap, the present work systematically investigates how the characteristics of recommended products or actions influence the effectiveness of LLM-generated personality-tailored persuasion. Across three studies, we employ a stepwise design that progresses from relatively open- ended, trait-consistent prompting—i.e., instructing the LLM to generate messages aligned with a specified Big Five trait—to increasingly constrained generation grounded in topic-central features. We focus on two theoretically grounded questions: (a) whether semantically anchoring prompts by pre-specifying the core features of the recommended product or action helps stabilize personality-matching effects, and (b) whether topic-specific stereotypes systematically influence the relative persuasiveness of different personality framings. By answering these questions, the current research seeks to identify key boundary conditions and influential factors governing LLM-driven personality-tailored persuasion, offering new insights into how such models may be more reliably leveraged for persuasive communication in broad domains. Building on this foundation, we propose two hypotheses.

H1: Fixing the core features of the recommended product or action in the prompt facilitates the emergence of personality-matching effects.

H2: Topic-specific stereotypes influence how audiences respond to personality-tailored persuasive messages.

## 2 Study 1

Study 1 was designed to examine whether personality-tailored messages generated from previously validated prompts can produce reliable personality-matching effects, when the content of the persuasive messages were not further controlled.

### 2.1 Materials and methods

In Study 1, we recruited 244 participants through Wenjuanxing, the largest online survey platform in China. The inclusion criteria were: (1) age between 18 and 60 years; (2) fluency in Chinese; and (3) provision of informed consent. The exclusion criterion was a self-reported history of any psychiatric disorder. All eligibility criteria were assessed based on self-report due to the online nature of the study. Prior to data analysis, 55 participants were excluded from analyses due to failure to pass at least one of two attention-check items, resulting in a final analytic sample of 189 participants. The mean age of participants was 23.89 years (*SD* = 6.06), and the sample was approximately balanced by gender (92 males, 97 females). Most participants (95.8%) reported having a bachelor’s degree or higher.

Using the GPT-4 API (version gpt-4-1106-preview) with temperature set to 0.7 to allow stylistic variation and seed fixed at 1234 to ensure reproducibility, we generated personality-tailored persuasive messages for two representative topics: influenza vaccination (public health) and smartwatches (consumer product). Each topic was paired with two Big Five traits—Openness to Experience and Conscientiousness—at both high and low levels, yielding eight messages in total. We manipulated one trait at a time to ensure analytical clarity in isolating the focal mechanisms of interest. Manipulating multiple traits simultaneously would substantially complicate interpretation, as null effects could stem from ineffective trait-level personalization, cross-trait interactions, or attenuation by topic stereotypes. Examples of the prompts and model-generated messages for the smartwatch condition are shown in **Table 1** (see **Supplementary Material**, Section 1.2 for the complete set of prompts and generated messages.) The prompting framework followed the structured, role-based approach proposed by Xu and Zhao (39), which employs personality cues to guide message generation while instructing the model to avoid explicitly referencing these traits in the output.

**Table 1:** Study 1: Example prompts and model-generated messages for the smartwatch condition.

Targeted trait	Prompt	Personalized message
High Openness	As a senior advertising specialist, you create persuasive, personalized ads for specific audiences. Generate an advertisement about smartwatches for people who are open to new experiences and artistic. Write one paragraph of no more than 100 words, using arguments, tone, and style that fit this audience. Please always follow these rules: Although you use personality characteristics to guide your generation strategy, do not directly reference these details in the ad. The audience should not know you possess this information, nor should they be able to tell that the ad is personalized. Please respond in Chinese.	Discover the art of living, right on your wrist. This smartwatch is not only a timepiece of art but also a creative extension of your individuality. Every gesture and moment of inspiration is seamlessly captured, sparking new ideas and supporting your explorations. With customizable watch faces that reflect your unique style and thoughtfully delivered notifications, the device integrates creativity into everyday life. Let time meet aesthetics—explore freely and create without limits.
Low Openness	As a senior advertising specialist, you create persuasive, personalized ads for specific audiences. Generate an advertisement about smartwatches for people who are down-to-earth and traditional. Write one paragraph of no more than 100 words, using arguments, tone, and style that fit this audience. Please always follow these rules: Although you use personality characteristics to guide your generation strategy, do not directly reference these details in the ad. The audience should not know you possess this information, nor should they be able to tell that the ad is personalized. Please respond in Chinese.	This smartwatch is practical and efficient, making everyday routines easier to manage. With one-tap access to health data, calls, and payments, you can quickly handle essential tasks without complicated settings. Designed for those who value stability and functionality, it brings convenience without unnecessary flashiness. Choose a smartwatch to enjoy a simpler, more effortless life.

To enrich the personality cues within this framework, we incorporated trait descriptors inspired by Matz et al. (37)’s application of the Ten-Item Personality Inventory (TIPI; (49)) in personalized persuasion research. Specifically, GPT-4 was prompted to write messages for individuals described as open to new experiences and artistic versus down-to-earth and traditional (Openness), and as dependable and organized versus disorganized and careless (Conscientiousness). This descriptor-based strategy preserved the original prompt structure while providing clearer linguistic cues.

To verify that the generated messages conveyed the intended personality orientations, ten graduate students majoring psychology independently classified each stimulus as reflecting high or low Openness or Conscientiousness. Expert agreement reached 100%, indicating that the personality framings were unambiguously identifiable.

After providing informed consent, participants read paired messages within each topic, with the presentation order of high- and low-trait versions randomized to control for order effects. After each pair, participants evaluated the two messages comparatively on three dimensions: perceived persuasiveness (“This message is persuasive”), attitude change (“After reading this message, I feel more positive toward the topic”), and behavioral inclination (“After reading this message, I am more likely to take the suggested action”). Following (37), each comparison employed an 11-point bipolar scale ranging from 1 (“Message A is more persuasive”) to 11 (“Message B is more persuasive”), with the midpoint of 6 indicating that both messages were perceived as equally persuasive. The bipolar format minimized response substitution bias Graham and Coppock (50) and provided a direct measure of participants’ relative evaluations of message effectiveness.

Participants then completed the Chinese version of the Big Five Inventory–44 (BFI–44; (51)) to assess their personality traits on a 5-point Likert scale ranging from 1 (strongly disagree) to 5 (strongly agree). Internal consistency was acceptable for the focal dimensions of Openness (*α* = .77) and Conscientiousness (*α* = .85).

To test the personality-matching hypothesis (H1), participants were categorized into high and low groups for each focal trait using a median-split approach. This approach is a commonly used and broadly accepted analytical practice in psychological research (52–55), enabling a clear test of the theoretically specified personality-matching hypotheses. Participants whose scores fell exactly at the median were excluded from the corresponding trait analysis to ensure clear group differentiation. To examine topic stereotype effects (H2), one-sample tests were used to assess whether overall message preferences (including all participants) deviated from the scale midpoint. All statistical tests were two-tailed with an alpha level of .05, and false discovery rate (FDR) correction was applied to account for multiple comparisons.

### 2.2 Results and discussion

**Figure 1:**
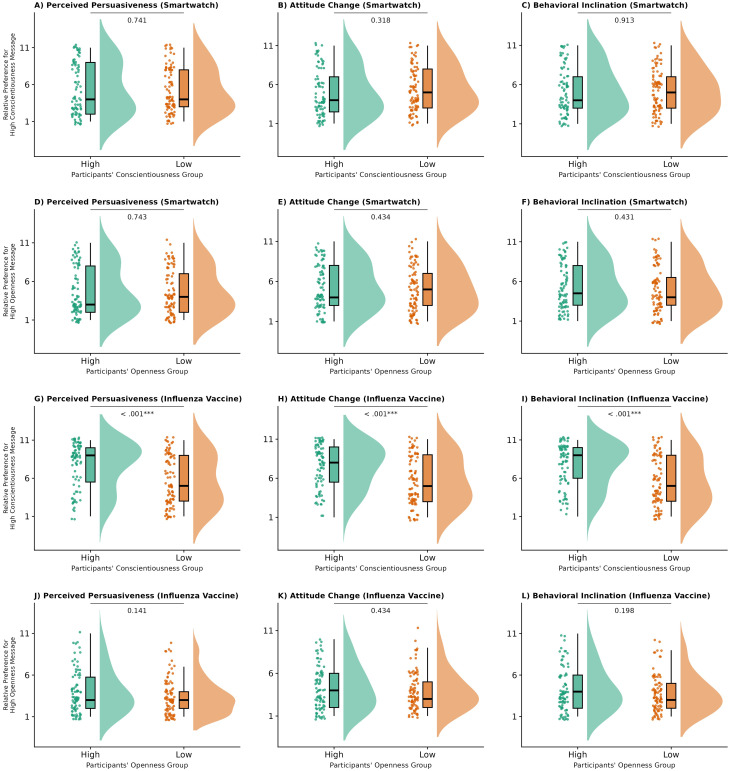
Effects of trait-message congruence on persuasion outcomes in Study 1. Raincloud plots display distributions, individual data points, and boxplots for high-trait participants (green) versus low-trait participants (orange) across different message topics and personality traits. **(A–F)** show smartwatch messages; **(G–L)** show influenza vaccine messages. P-values from independent samples t-tests comparing preference for high-trait messages between groups are displayed above each comparison. *p<.05, **p<.01, ***p<.001.

To test the personality-matching effect, participants were median-split on the focal traits, and independent-samples t tests were conducted on the high-trait message preference scores for each condition. **Figure 1** displays the distributions and group comparisons across all trait–topic combinations; detailed statistics are reported in **Supplementary Material Table S4**. After false discovery rate (FDR) correction, a significant matching effect emerged only for the influenza-vaccine messages tailored to Conscientiousness. Participants high in Conscientiousness showed stronger preferences for the high-Conscientiousness message than did low Conscientiousness participants on all three outcomes: perceived persuasiveness, t(173.75) = 4.38, p < .001, d = 0.66; attitude change, t(173.98) = 4.28, p < .001, d = 0.64; and behavioral inclination, t(173.98) = 4.79, p < .001, d = 0.72. No significant effects were observed for any other trait–topic combinations.

To assess the possible effects of topic stereotype, one-sample t tests compared participants’ mean preference scores for each condition against the midpoint 6 of the 11-point scale, where scores above 6 indicated a preference for the high-trait message(see **Supplementary Table S5** in the **Supplementary Material** for full results). In the smartwatch topic, preference scores for messages tailored to Openness and Conscientiousness were significantly below the midpoint across all outcomes (all ps < .001, ds ≈ 0.24–0.43), indicating an overall advantage for low-Openness and low-Conscientiousness messages. In contrast, in the influenza-vaccine topic, high-Conscientiousness messages showed a small but reliable advantage in perceived persuasiveness, t(188) = 2.24, p = .026, d = 0.16, whereas low-Openness messages were strongly preferred across all outcomes (all ps < .001, ds ≈ 0.82–1.01). These patterns remained significant after FDR correction. When controlling for demographic variables including age and gender, the overall pattern of results remained consistent, providing additional evidence for the robustness of the observed effects.

Study 1 examined whether personality-tailored messages generated from previously validated prompts could produce reliable matching effects when the substantive features of the message content were not controlled. The expected matching patterns did not consistently emerge, with a significant effect appearing only in the Conscientiousness × influenza vaccination condition. To better understand this inconsistency, we conducted qualitative inspections of the generated messages, and revealed notable variation in how different personality framings represented the product or action’s essential features. For instance, in the smartwatch condition (**Table 1**), the low-Openness message foregrounded concrete utilities such as health monitoring and mobile payment (e.g., “one-tap access to health data, calls, and payments”), whereas the high-Openness message shifted toward symbolic and experiential themes (e.g., “a creative extension of your individuality” and “a timepiece of art”). Although stylistically appropriate, the latter paid comparatively little attention to the functional benefits that typically anchor persuasion in this topic—consumers generally choose smartwatches for their core practical functions rather than their ability to express individuality. This example illustrates how, in the absence of content constraints, personality-tailored messages can diverge in semantic focus. Such divergence may weaken argument strength and help explain why personality-matching effects were difficult to detect.

In addition to these inconsistencies, the results also showed cross-group preference. In the smartwatch topic, low-Openness and low-Conscientiousness framings were generally preferred, whereas in the influenza-vaccine topic, high-Conscientiousness and low-Openness framings received broader endorsement. Given that personality matching effects were not consistently detected, it remains unclear whether these audience-wide preferences reflect topic stereotypes or result from unsuccessful personality tailoring. If personality-tailored messages inadequately conveyed the core features of the recommended product or action, this could undermine their persuasiveness and similarly produce such generalized preferences. Further experimentation is needed to distinguish between these potential sources.

Taken together, the findings of Study 1 suggest that constraining the core features of the recommended product or action may be a useful—and perhaps necessary—step for enabling LLMs to generate effective personality-tailored persuasive messages. Future research should test the efficacy of this approach and further examine whether stereotype-consistent baseline preferences reliably emerge across topics.

## 3 Study 2

Building on the findings of Study 1, Study 2 tested whether anchoring the core features of the recommended product or action would enable personality-tailored persuasive messages to produce stable personality-matching effects.

### 3.1 Materials and methods

Study 2 replicated the procedure of Study 1 with one key methodological improvement: semantic anchoring. Prior LLM-based personality-tailored persuasion research has typically allowed free message generation, which may simultaneously vary both substantive content and expressive style (37, 38). To control for substantive content and isolate stylistic variations, the high- and low-trait messages were constrained to convey identical core features of the recommended product or action, ensuring that the observed personality-targeted expressions did not alter the core points of the message. For example, in the smartwatch condition, we added the instruction “highlight concrete daily-management functions such as health monitoring, answering calls, and mobile payment” to the prompt, thereby fixing the key product features communicated across all generated persuasive messages (**Table 2**; see **Supplementary Material**, Section 2.2 for all materials).

**Table 2:** Study 2: example prompts and model-generated messages for the smartwatch condition.

Targeted trait	Prompt	Personalized message
High Openness	As a senior advertising specialist, you create persuasive, personalized ads for specific audiences. Generate an advertisement about smartwatches for people who are open to new experiences and artistic. Write one paragraph of no more than 100 words, using arguments, tone, and style that fit this audience. Highlight concrete daily-management functions such as health monitoring, answering calls, and mobile payment. Keep the content substantive and avoid overly flashy language. Please always follow these rules: Although you use personality characteristics to guide your generation strategy, do not directly reference these details in the ad. The audience should not know you possess this information, nor should they be able to tell that the ad is personalized. Please respond in Chinese.	Explore the art of living—this smartwatch brings color and creativity to your day. Whether it’s tracking your health metrics during a workout, answering an important call with a single tap, or completing a payment at a café with a flick of the wrist, it is an indispensable partner in life. Its refined craftsmanship and cutting-edge technology make everyday management more elegant and expressive. Add intelligence and convenience to your routine, making each day not only organized but also full of artistry.
Low Openness	As a senior advertising specialist, you create persuasive, personalized ads for specific audiences. Generate an advertisement about smartwatches for people who are down-to-earth and traditional. Write one paragraph of no more than 100 words, using arguments, tone, and style that fit this audience. Highlight concrete daily-management functions such as health monitoring, answering calls, and mobile payment. Keep the content substantive and avoid overly flashy language. Please always follow these rules: Although you use personality characteristics to guide your generation strategy, do not directly reference these details in the ad. The audience should not know you possess this information, nor should they be able to tell that the ad is personalized. Please respond in Chinese.	In a busy daily routine, this smartwatch becomes a reliable companion. It not only tracks your health with precision—monitoring heart rate and steps throughout the day—but also lets you answer important calls without reaching for your phone. Its convenient payment function makes everyday shopping effortless. With the classic look of a traditional watch and the ease of modern technology, this smartwatch brings a simple yet meaningful upgrade to your life.

We recruited 269 participants through Wenjuanxing. The inclusion and exclusion criteria were identical to those in Study 1. Prior to data analysis, participants who failed at least one of two attention-check items were excluded ( n=50), yielding a final analytic sample of 219 participants (116 males, 103 females; $M_{\text{age}}$ = 26.37, SD = 4.21). Most participants (99.1%) held a bachelor’s degree or higher.

All stimuli in this study were pre-validated using the same expert-judgment procedure employed in Study 1, ensuring that each message clearly expressed its intended personality orientation.

As in Study 1, participants evaluated paired messages on an 11-point bipolar scale assessing relative persuasiveness, attitude change, and behavioral inclination. They then completed the Chinese BFI-44 (51), which demonstrated good internal consistency for Conscientiousness (*α* = .90) and Openness (*α* = .78).

Data analysis procedures followed the same approach as Study 1, using median-split categorization (excluding median scores) to test personality-matching effects and one-sample tests to examine topic stereotypes, with FDR correction applied.

### 3.2 Results and discussion

**Figure 2:**
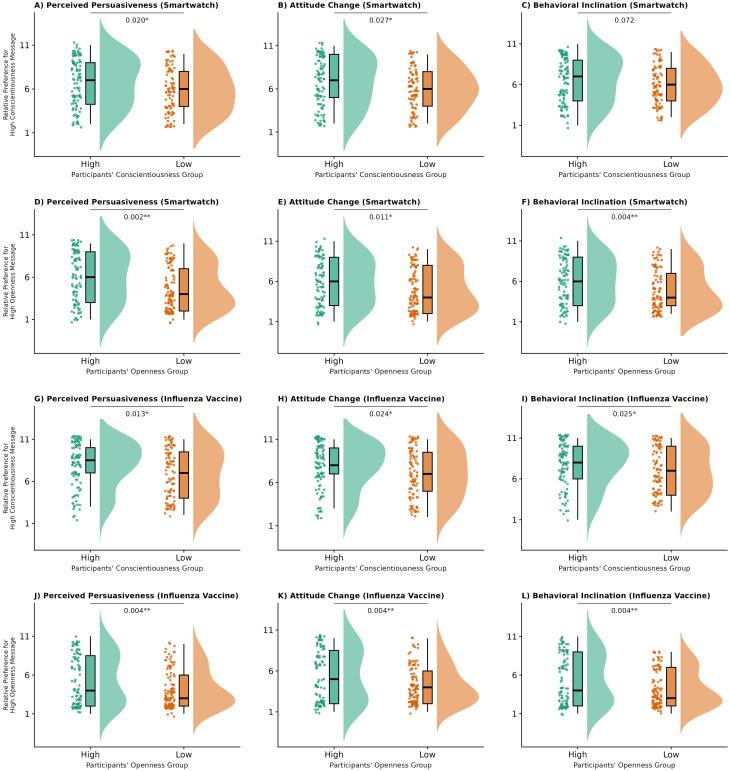
Effects of trait-message congruence on persuasion outcomes in Study 2. Raincloud plots display distributions, individual data points, and boxplots for high-trait participants (green) versus low-trait participants (orange) across different message topics and personality traits. **(A–F)** show smartwatch messages; **(G–L)** show influenza vaccine messages. P-values from independent samples t-tests comparing preference for high-trait messages between groups are displayed above each comparison. *p<.05, **p<.01, ***p<.001.

Compared with Study 1, where the personality-matching effect was limited to the vaccine–Conscientiousness condition, Study 2 revealed a broader pattern once message content was standardized (**Figure 2**; detailed statistics in **Supplementary Material Table S9**). Independent-samples t tests showed significant personality-matching effects across nearly all conditions ( ps < .05, ds = 0.32–0.45). The smartwatch–Conscientiousness condition also exhibited a small effect in the predicted direction ( d = 0.26), although the associated t test did not reach statistical significance, t(217) = 1.80, p = .072.

One-sample t tests further confirmed cross-group preference for messages framed according to specific personality traits (see **Supplementary Table S10** in the **Supplementary Material** for full results). For the smartwatch topic, participants showed an overall preference for low-Openness messages ( ts(218) = −2.81 to −2.59, ps < .010, ds = 0.17–0.19). For the vaccine topic, participants showed overall preferences for both low-Openness ( ts(218) = −6.32 to −6.02, ps < .001, ds = 0.41–0.43) and high-Conscientiousness messages ( ts(218) = 7.07–7.25, ps < .001, ds = 0.48–0.49). All effects remained significant after FDR correction and were unaffected by demographic controls.

Study 2 tested H1 by examining whether fixing the core features of the recommended product or action would allow LLM-generated personality-matching effects to emerge more reliably. The results strongly supported this hypothesis: once semantic drift was minimized, stylistic variations aligned with personality traits produced stable matching effects across topics. Building on this foundation of successful personality tailoring and consistent matching effects, the cross-group preferences observed in Study 2 are more plausibly interpreted as stable, stereotype-consistent preference patterns. For instance, low-Openness framings for smartwatches were rated more favorably overall—likely reflecting the perception of smartwatches as primarily functional, utility-oriented devices. Likewise, high-Conscientiousness framings for influenza vaccination received higher ratings across recipients, consistent with the widely shared view of vaccination as a responsible, duty-oriented health behavior. These tendencies parallel the topic stereotypes identified in Study 1, suggesting that such expectations can shape how audiences respond to the messages independently of their own personality traits, even when semantic content is held constant.

As in Study 1, Study 2 focused on two representative persuasive topics from public health and consumer domains—receiving an influenza vaccine and purchasing a smartwatch—and examined two personality traits: Conscientiousness and Openness. Important questions remain for future research: Does semantic anchoring remain effective for LLM-based personality customization across other topics and traits? Do topic stereotype effects persist across a broader range of persuasion contexts? Further empirical investigation is needed to address these questions and establish the generalizability of the present findings.

## 4 Study 3

Study 3 was designed to examine whether topic-specific stereotypes systematically shape responses to personality-tailored persuasive messages across a broader set of topics (H2), while also providing a further test of whether personality-matching effects could be stably achieved in broader conditions when message content is anchored to the core features of the recommended product or action (H1).

### 4.1 Materials and methods

Study 3 employed the same design and procedure as Study 2, extending the paradigm to include multiple topics and additional personality traits. We recruited 269 participants through Wenjuanxing. The inclusion and exclusion criteria were identical to those in Study 1. Priorto data analysis, participants who failed at least one of two attention-check items were excluded ( n=59), yielding a final analytic sample of 210 participants. The final sample (102 males, 108 females; $M_{\text{age}}$ = 24.18, SD = 2.79) was highly educated (99.5% held a bachelor’s degree or higher).

Five persuasive topics recommending different products or actions—each likely reflecting prototypical personality-related stereotypes—were identified through *a priori* consensus among three psychologists with expertise in personality and social psychology, who independently reviewed the topics and agreed on their associations with the Big Five traits. The selected topics included: photography workshops (high Openness), Pop Mart blind boxes (low Conscientiousness), Strawberry Music Festival (high Extraversion), disaster relief donations (high Agreeableness), and C’estbon bottled water (neutral). For each stereotype-associated topic, GPT-4 generated both high- and low-trait messages; for the neutral topic, high- and low-trait versions were created for Openness and Conscientiousness, yielding 12 messages in total (see **Supplementary Material**, Section S3.2 for all prompts and generated messages).

Message generation followed the same prompting framework as in Study 2, using semantic anchoring to ensure content equivalence across conditions. The core features defined by subject-matter experts included: creative use of light, composition aesthetics, and artistic vision development for photography workshops; surprise, trendy collectibles, and effortless enjoyment through the blind box mechanism for Pop Mart blind boxes; diverse atmosphere, open and free environment, and immersive musical experience for the music festival; post-disaster recovery support and concrete assistance that brings hope to affected communities for donations; and pure water quality, refreshing taste, and reliable health and safety for bottled water. All stimuli were pre-validated following the same expert-judgment procedure as in Study 1 to confirm personality-trait clarity.

Measures and procedures were identical to those in Study 2. Participants compared paired high- and low-trait messages on three 11-point bipolar scales assessing perceived persuasiveness, attitude change, and behavioral inclination (37, 50). They then completed the Chinese BFI–44 (51) assessing Openness ( α=.83), Conscientiousness ( α=.86), Extraversion ( α=.85), and Agreeableness ( α=.86).

Data analysis followed the same procedures as in Studies 1 and 2. Median-split categorization (excluding participants at the median) was used to test personality-matching effects, and one-sample *t* tests examined topic stereotype effects across all participants.

### 4.2 Results and discussion

**Figure 3:**
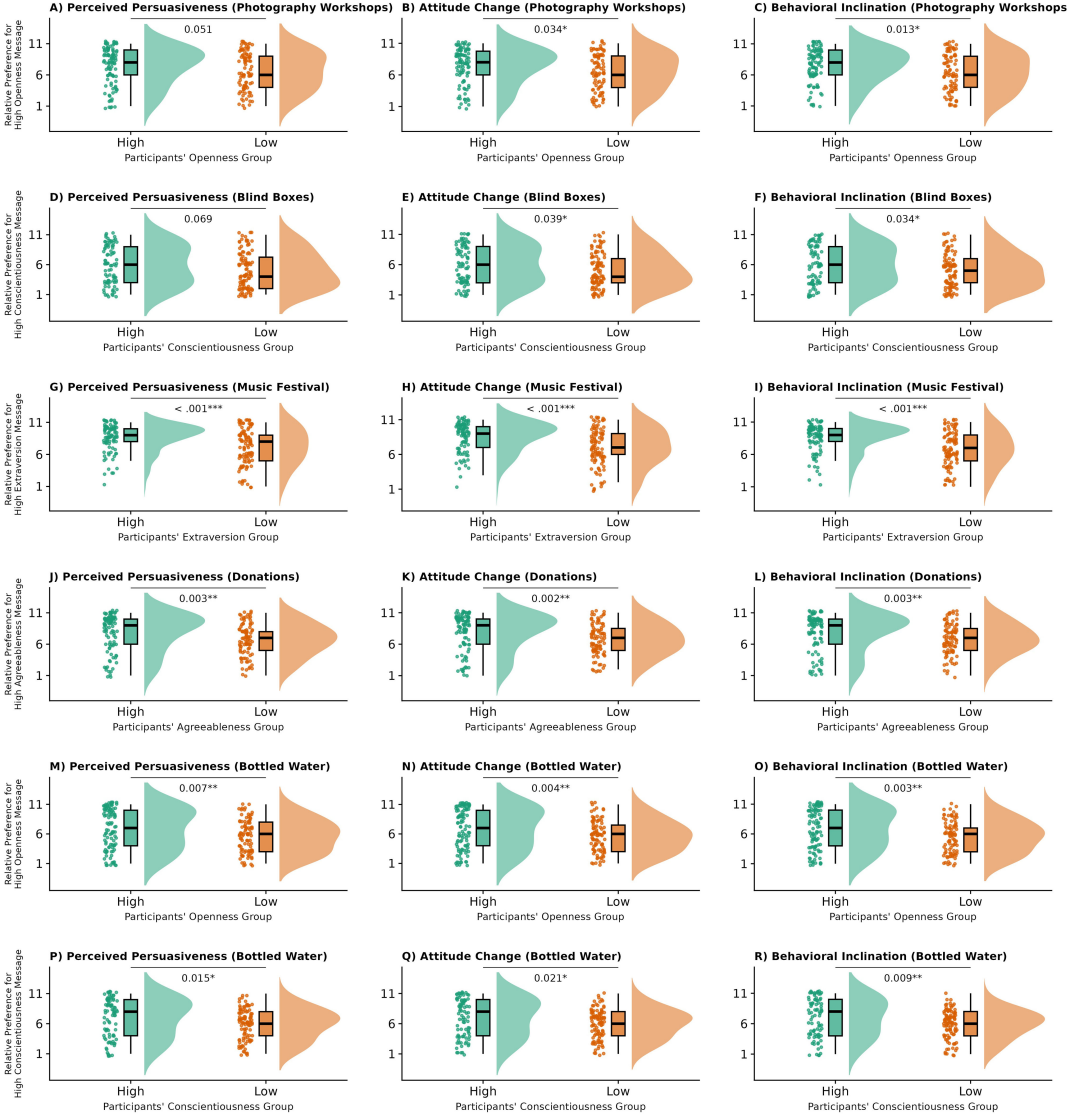
Effects of trait-message congruence on persuasion outcomes in Study 3. Raincloud plots display distributions, individual data points, and boxplots for high-trait participants (green) versus low-trait participants (orange) across different message topics and personality traits. **(A–C)** show photography workshop messages; **(D–F)** show blind box messages; **(G–I)** show music festival messages; **(J–L)** show donation messages; **(M–R)** show bottled water messages. P-values from independent samples t-tests comparing preference for high-trait messages between groups are displayed above each comparison. *p<.05, **p<.01, ***p<.001.

Independent-samples *t* tests revealed significant personality-matching effects in 16 of the 18 experimental conditions ( ps < .05), all of which remained significant after FDR correction (**Figure 3**; detailed statistics in **Supplementary Material Table S14**). Effect sizes ranged from d=0.27 to 0.68, indicating overall medium effects. The two nonsignificant conditions exhibited the same directional pattern.

One-sample *t* tests were conducted to examine the effects of topic stereotype, testing whether message preferences significantly differed from the scale midpoint 6 (see **Supplementary Table S15** in the **Supplementary Material** for full results). 13 of the 18 conditions were significant ( ps < .05) and remained so after FDR correction. Effect sizes ranged from *d* = 0.18 to 0.74, suggesting that certain personality-tailored messages demonstrated persuasive advantages across audiences. All four topics theoretically associated with specific personality traits—including photography workshops (high Openness), music festivals (high Extraversion), disaster donations (high Agreeableness), and blind boxes (low Conscientiousness)—showed significant preferences consistent with theoretical predictions. Specifically, high-Openness messages for photography workshops were rated more favorably ( ts(209) = 4.48–5.00, ps < .001, ds = 0.31–0.35), as were high-Extraversion messages for music festivals ( ts(209) = 9.37–10.67, ps < .001, ds = 0.65–0.74) and high-Agreeableness messages for disaster donations ( ts(209) = 7.09–7.56, ps < .001, ds = 0.49–0.52), whereas the blind box topic favored low-Conscientiousness messages ( ps < .05, d=0.18– 0.20). In contrast, message preferences for bottled water topic did not deviate from the midpoint (all |d|< 0.20), consistent with its theoretically neutral nature. After controlling for demographic covariates (age and gender), the overall pattern of results remained unchanged, confirming the robustness of both personality-matching and topic stereotype effects.

Study 3 extended the examination to a broader set of topics and personality traits and asked whether the patterns observed in the earlier studies would generalize. Across this wider range, we again found stable personality-matching effects under semantically anchored prompts, further corroborating H1. At the same time, clear stereotype-consistent preference patterns emerged: framing styles such as high-Openness wording for photography workshops, high-Extraversion wording for music festivals, high-Agreeableness wording for disaster-relief donations, and low-Conscientiousness wording for blind boxes received more favorable evaluations across recipients 378 regardless of their own personalities. These topic stereotype effects indicate that message effectiveness is shaped not only by personality congruence with the recipient but also by the degree to which a framing style fits widely shared expectations about the topic, providing strong support for H2.

## 5 General discussion

Across three studies, the present research systematically examined how the effectiveness of LLM-generated personality-tailored persuasion is shaped by content constraints and topic-specific expectations. Study 1 showed that when the core features of the recommended product or action were not controlled, some personality-targeted versions drifted away from the topic’s functional center, and the resulting cross-version variability in argument strength contributed to limited and inconsistent personality-matching effects. At the same time, Study 1 revealed cross-group preferences, though it remained unclear whether these reflected topic stereotypes or resulted from unsuccessful personality tailoring.

Study 2 addressed the content-drift problem by anchoring the core features of the recommended product or action. Under these semantically aligned conditions, robust personality-matching effects emerged across multiple traits and topics, providing clear support for H1. Study 2 also provided clear evidence of topic stereotype effects, demonstrating that topic-specific expectations systematically influence the persuasive impact of messages—independently of recipient personality—even when semantic content is held constant.

Study 3 extended the investigation to a broader and more heterogeneous set of topics. The persistence of personality-matching effects across nearly all conditions demonstrates that semantic anchoring could stabilize these effects in broader persuasive contexts, offering additional confirmation of H1. Crucially, these observed personality-matching effects reflect systematic tendencies across relative personality levels within our samples. At the same time, Study 3 showed that the stereotype effects appear to be a pervasive phenomenon in LLM-generated personality-tailored persuasion, offering strong and convergent evidence for H2.

### 5.1 Why semantic anchoring stabilizes personality-matching effects

A central finding of this work is that personality-matching effects emerged more reliably when message content was anchored to the core features of the recommended product or action. This result suggests that semantic anchoring operates as a methodological safeguard against LLM-specific semantic drift, helping to ensure that observed matching effects are more likely to reflect stylistic congruence rather than unintended variation in argument quality. In contrast, without such anchoring, trait-targeted message variants may differentially emphasize core benefits versus peripheral appeals, leading to inconsistent argument strength that obscures matching effects. This key improvement to the standard practice in prior LLM-based personality-tailored persuasion research (37, 38) offers an innovative way to better leverage the message generation capabilities of large language models.

Mechanistically, this drift may stem from how LLMs interpret and expand trait-descriptive prompts. The TIPI adjective pairs used in our instructions—such as “open to new experiences and artistic” versus “down-to-earth and traditional” for Openness or “dependable and organized” versus “disorganized and careless” for Conscientiousness (49)—function as compact semantic cues. Rather than treating these cues as markers of deeper motivational orientations, LLMs process them primarily as stylistic signals. Prior work shows that LLMs rely heavily on pattern completion and surface-level linguistic markers (56, 57). Thus, when prompted with adjectives such as “artistic” or “dependable,” the model expands these terms through highly patterned expressions (e.g., “explore the unknown,” “discover life’s artistry,” “pursue excellence,” “enhance efficiency”) that recur across topics regardless of their functional demands. This pattern-matching process reliably produces stylistic alignment with the trait cues but does not ensure that the content meaningfully engages with the core features of the recommended product or action.

Crucially, nothing in this process provides a mechanism for linking personality traits to the motivational routes or argument structures that would be persuasive for a given behavior. TIPI descriptors do not specify which concrete concerns, benefits, or facilitators are most compelling for people high versus low in a trait. Correspondingly, current LLMs lack an inference system capable of constructing a structured chain such as trait cue → motivational orientation → functionally strong arguments.

Instead, they rely on unambiguous stylistic markers—such as symbolic phrases like “timepiece of art” to signal Openness, or functional terms like “efficient” and “excellence” for Conscientiousness—to represent personality. This reliance helps explain why the generated messages were easily identifiable by expert raters (Study 1), yet did not yield consistent persuasive effects. In the absence of such a mapping, generation is guided by semantic proximity rather than by psychologically meaningful personality–behavior linkages.

By contrast, human-authored personalization explicitly fills in this missing layer. In Hirsh et al. (25), designers first identified the motivational priorities theoretically associated with each Big Five trait—for example, order, efficiency, and achievement for Conscientiousness, or creativity, novelty, and intellectual exploration for Openness—and then crafted arguments that expressed these priorities while preserving coherence with the product’s core features. The resulting messages differed in motivational framing but remained anchored to the topic’s functional benefits, ensuring both relevance and persuasive adequacy.

Consequently, while human-authored messages achieve motivational alignment without compromising argument strength, unconstrained LLM outputs tend to exhibit content drift and stylistic templating. The persuasive materials used in our studies illustrate this mechanism clearly. Conceptually, smartwatches are typically positioned as functional products that solve practical problems—monitoring health data, managing notifications, and enabling mobile payments. Yet the LLM-generated messages diverged in how these core features were represented: the low-Openness version foregrounded these practical utilities, whereas the high-Openness version, driven by cues such as “open to new experiences” and “artistic,” shifted toward symbolic and experiential themes with limited attention to essential functions. This semantic drift moves the message away from the functional script that usually anchors persuasion in this category, weakening argument strength and making congruence less consequential. In line with evidence that matched messages are persuasive only when they offer strong, functionally relevant arguments—because motivational congruence encourages more systematic processing of the content (58)—such stylistically personalized but substantively weak messages may fail to produce personality-matching effects.

Anchoring the core features of the recommended product or action in the prompt directly targets this problem. By first specifying the functional backbone of the message—such as health monitoring, call management, mobile payment for smartwatches, or disease prevention, personal protection for vaccination—all generated messages are forced to cover the same set of core benefits and risks, regardless of personality framing. Within this constrained semantic space, the trait descriptors primarily modulate how these shared elements are presented: high-Openness messages can describe mobile payment as “completing a payment with a light touch at a café corner,” whereas low-Openness messages can stress “a convenient payment feature that handles everyday shopping with ease”; high-Conscientiousness messages can frame vaccination as “a responsible, scientifically grounded way to prevent flu and protect both your own health and the health of others,” whereas low-Conscientiousness messages can emphasize “preventing flu with a quick shot so you can avoid unnecessary trouble in your daily routine.” In short, content anchoring reduces variance in argument quality and topic relevance, allowing personality-congruent stylistic cues to operate on a more even baseline.

### 5.2 Topic stereotypes: a key determinant of personality-tailored persuasion

Across studies, we also observed a second form of systematic variation in message effectiveness—one that arises not from individual differences but from the shared social meaning associated with each persuasive topic. We refer to this pattern as the topic stereotype effect. By “topic stereotypes,” we mean the culturally shared expectations about how a given topic is typically talked about, and what motivational or stylistic orientation “fits” the topic (59). These expectations often resemble personality-like traits along the Big Five dimensions. For instance, disaster relief donations are widely associated with warmth, care, and prosocial concern; and music festivals are linked to energy, sociability, and excitement. When the stylistic framing of a message aligns with these socially shared stereotypes, the message tends to be perceived as more appropriate and compelling—even when such framing conflicts with the recipient’s own personality profile.

Several psychological mechanisms help explain why topic–message congruence produces reliable persuasive benefits. First, different topics carry specific functional scripts: health topics emphasize safety and responsibility, while experiential topics emphasize novelty and exploration. When messages align with these scripts using matching personality styles, audiences experience a sense of appropriateness—a “feels right” effect consistent with regulatory fit theory (60, 61). Second, topic stereotypes function as shared schemas that shape expectations about how topics should be presented. Schema-congruent messages are processed more fluently, and this ease enhances evaluations (59, 62). This also explains why neutral topics show no overall advantage: without alignable schemas, stylistic cues cannot generate fluency gains. Third, for value-laden topics such as prosocial or risk-management issues, messages framed with high Agreeableness or Conscientiousness resonate more strongly with societal value frameworks, enhancing both legitimacy and relevance (63–66). Finally, cultural value frameworks may amplify certain stereotypes. In Chinese contexts, for example, public health and collective welfare topics are evaluated through strongly normative lenses, which may further elevate the persuasiveness of high-Conscientiousness or high-Agreeableness framings (67, 68).

The origins of the topic stereotype effect are fundamentally distinct from those of personality-matching effects. Personality-matching effects arise from individual differences—namely, recipients’ differential preferences aligned with their trait dispositions, whereas topic stereotype effects stem from shared contextual schemas—culturally shaped expectations about how certain topics are typically discussed. These schemas apply uniformly across the entire audience, meaning that stylistic alignment benefits all recipients rather than a trait-defined subset. Consequently, topic stereotype effects often exert broader influence on persuasive outcomes. Indeed, across our studies, the effect sizes associated with topic stereotypes were often larger than those for personality matching, underscoring the substantial and non-negligible role of topic stereotypes in personality-tailored persuasion.

### 5.3 Practical implications

The present findings also clarify how topic content can be operationalized in applied LLM-based personalization systems. Anchoring the core features of the recommended product or action provides a practical solution to the semantic drift that often arises when LLMs over-extend trait-descriptive cues. In real-world applications—such as product promotion, public-service messaging or mental health communication—designers can treat content anchoring as a structural scaffold: by first articulating the topic’s essential benefits, risks, and use-cases, they ensure that all generated variants share a coherent argumentative foundation. Within this stabilized semantic space, personality cues can then adjust tone and stylistic framing without altering the message’s functional meaning. This content-anchored personalization approach offers a feasible and scalable pathway for deploying personality-tailored persuasion with current-generation LLMs, which excel at stylistic modulation but require structural guidance to maintain motivationally grounded argumentation.

Furthermore, the interplay between topic stereotypes and personality matching offers potential guidance for strategic prioritization: When might practitioners invest in personality customization versus relying on stereotype-congruent messaging? Our findings suggest that the answer may depend on the strength of the stereotypes associated with the topic.

For topics with strong, culturally shared stereotypes (e.g., music festivals), our results indicate a general baseline preference for the stereotype-congruent style across the population. In such cases, practitioners may consider prioritizing stereotype-congruent messaging as a reasonable baseline to ensure stylistic legitimacy and reduce the risk of perceived inappropriateness. For instance, in mental health contexts, therapeutic communication is normatively anchored in a stereotype of “Warmth,” emphasizing empathy, emotional support, and non-judgment. By contrast, campaigns aimed at destigmatizing mental illness or promoting social welfare may align more naturally with stereotypes of “Responsibility” or “Competence,” highlighting civic duty and factual clarification. In these settings, personalization appears most beneficial for high-congruence segments, whereas for individuals whose traits conflict with the topic stereotype, adhering to the dominant normative style (e.g., maintaining Warmth in therapeutic messaging) is likely safer than aggressive personality tailoring, which may violate expectations of care and thereby undermine trust, perceived appropriateness, and adherence.

By contrast, when topics lack a strong, culturally shared stereotype (e.g., Bottled Water), where no dominant stylistic norm exists, personality customization may play a more central role. In the absence of a universally effective “default” style, LLM-driven tailoring may offer a viable path to persuasive effectiveness by adjusting the framing to resonate with divergent audience preferences. To implement this tiered approach, practitioners might consider gauging the baseline strength of topic stereotypes—potentially through pilot testing to detect dominant stylistic preferences or through exploratory analyses of historical engagement patterns—to inform whether personalization should serve as a primary consideration (in neutral contexts) or an incremental optimization.

At the same time, it is important to delineate the limits of this strategic framework for applied deployment, as the translational relevance of these insights may vary across domains that differ in consequentiality. For example, mental health communication spans a broad spectrum, ranging from relatively low-consequential contexts (e.g., public psychoeducation and the promotion of self-care behaviors) to more consequential situations (e.g., encouraging individuals to initiate, adhere to, or persist in a specific treatment). For the former, our findings offer relatively direct design guidance: content anchoring can ensure a coherent informational foundation, while adherence to topic-appropriate stylistic norms can help maintain perceived legitimacy and approachability. For the latter, however, effective personalization is likely to require a more holistic consideration of factors beyond audience personality alone, including clinical severity and risk, stage of change, and relational context. Accordingly, our results are best interpreted as most applicable to lowering the entry barrier for early-stage mental-health-related communication and scalable support, rather than as a standalone solution for the more complex, interactive dynamics characteristic of psychotherapy or treatment decision-making.

### 5.4 Limitations and future directions

The present study has several limitations that may inspire future work. First, with respect to measurement and analysis, the dependent measures relied primarily on self-reported persuasiveness and intentions, which are vulnerable to social desirability biases (69) and may not fully translate into actual behavior. Future research could strengthen external validity by incorporating behavioral field data, such as click-through or choice records. In addition, the use of median splits to categorize participants depends on the specific distribution of the current sample. While this approach captures relative congruency, supplementary analyses using continuous personality scores (see **Supplementary Tables S1**-**S3**, **S6**-**S8**, **S11**-**S13**) yielded consistent patterns, suggesting that the observed effects are not artifacts of dichotomization.

Second, technical constraints of current LLMs limited the complexity of our experimental manipulations. We manipulated one personality trait at a time because preliminary testing revealed that current models process multi-trait prompts additively rather than holistically (37), hindering integrated personality expression. While this single-trait approach facilitated clearer interpretation of focal mechanisms, multi-trait manipulations could be explored in future work as LLM capabilities continue to evolve. Relatedly, our reliance on concise trait descriptors (e.g., TIPI-based prompts) and semantic anchoring, while providing robust content stabilization, may limit the extent to which generated messages reflect deeper, motivation-based personality mechanisms beyond surface-level stylistic features. Future work could explore more sophisticated prompt architectures, such as chain-of-thought reasoning or plan-then-write workflows (70, 71), which may support the construction of more coherent arguments while preventing semantic drift and enabling more motivationally grounded forms of personalization. Additionally, it would be informative to benchmark LLM-generated personality-tailored messages against those crafted by human experts within the same experimental framework. Such head-to-head comparisons could help clarify the extent of the “automation gap” and the respective contributions of algorithmic scalability and human expertise in personality-tailored message design.

Third, regarding the identification of topic stereotypes in Study 3, we relied on expert consensus to establish theoretical links between topics and personality traits. While this approach ensured face validity, it did not incorporate independent empirical measurement from lay audiences prior to message generation. Future research could strengthen causal inference by adopting a two-stage design: first empirically identifying and validating topic stereotypes through norming studies (e.g., quantitative assessments of topic-linked personality perceptions), and then using verified topics to test their persuasive effects.

Finally, although the present stimuli covered diverse domains and provided an important foundation for LLM-supported mental health communication, investigation into specific mental health communication topics is highly necessary. We strongly recommend systematically incorporating mental health-relevant stimuli across diverse contexts in future research.

## Data Availability

The raw data supporting the conclusions of this article will be made available by the authors, without undue reservation.
