# Supplementary material for: How topic content shapes LLM personality-tailored persuasion: semantic anchoring and topic stereotype effects
Source: Front Psychiatry. 2026 Jan 30;17:1756792. doi: 10.3389/fpsyt.2026.1756792 (PMC12900667; doi:10.3389/fpsyt.2026.1756792)
Supplement: Supplementary file 1 [file DataSheet1.pdf]

Supplementary Material

1 STUDY 1

1.1 Additional Results

Table S1. Study 1: Means, Standard Deviations, and Pearson Correlations of Variables (Perceived Persuasiveness)

Variable	*M*	*SD*	1	2	3	4	5	6	7	8
1. Flu Vaccine × Openness	3.58	2.41								
2. Flu Vaccine × Conscientiousness	6.54	3.31	-.10							
3. Smartwatch × Openness	4.66	3.08	.35**	-.10						
4. Smartwatch × Conscientiousness	5.23	3.18	-.01	.11	-.10					
5. Openness	3.64	.56	.14	.20**	.06	-.01				
6. Conscientiousness	3.42	.69	.08	.22**	.07	.05	.24**			
7. Age	23.77	6.27	-.13	.08	-.12	.09	-.17*	.18*		
8. Gender	–	–	-.05	.04	.02	.17*	.11	.06	-.23**	

Note. * $p < .05$, ** $p < .01$.

Table S2. Study 1: Means, Standard Deviations, and Pearson Correlations of Variables (Attitude Change)

Variable	*M*	*SD*	1	2	3	4	5	6	7	8
1. Flu Vaccine × Openness	4.02	2.41								
2. Flu Vaccine × Conscientiousness	6.41	3.12	-.14							
3. Smartwatch × Openness	5.07	2.83	.34**	-.22**						
4. Smartwatch × Conscientiousness	5.15	2.87	-.15*	-.02	-.04					
5. Openness	3.64	.56	.11	.19*	.03	-.10				
6. Conscientiousness	3.42	.69	.03	.28**	-.02	-.04	.24**			
7. Age	23.77	6.27	-.15*	.13	-.16*	.11	-.17*	.18*		
8. Gender	–	–	-.06	-.01	.03	.12	.11	.06	-.23**	

Note. * $p < .05$, ** $p < .01$.

Table S3. Study 1: Means, Standard Deviations, and Pearson Correlations of Variables (Behavioral Inclination)

Variable	*M*	*SD*	1	2	3	4	5	6	7	8
1. Flu Vaccine × Openness	3.96	2.44								
2. Flu Vaccine × Conscientiousness	6.44	3.23	-.07							
3. Smartwatch × Openness	5.08	2.93	.34**	-.06						
4. Smartwatch × Conscientiousness	5.18	2.90	-.03	.03	.02					
5. Openness	3.64	.56	.13	.17*	.04	-.17*				
6. Conscientiousness	3.42	.69	.07	.28**	.04	.01	.24**			
7. Age	23.77	6.27	-.12	.11	-.13	.13	-.17*	.18*		
8. Gender	–	–	-.06	-.00	.08	.02	.11	.06	-.23**	

Note. * $p < .05$, ** $p < .01$.

Table S4. Study 1: Independent Sample *t*-Test Results for Trait–Message Congruence Effects

**Topic**	**Trait Condition**	**Outcome Measure**	*t*	*df*	*p*	p_{FDR}	*d*
Smartwatch	Conscientiousness	Perceived Persuasiveness	0.3308	164.31	0.7412	0.8104	0.0503
		Attitude Change	-1.0017	168.18	0.3179	0.5781	0.1519
		Behavioral Inclination	-0.1089	168.51	0.9134	0.9134	0.0165
	Openness	Perceived Persuasiveness	0.3285	172.11	0.7429	0.8104	0.0495
		Attitude Change	0.7848	173.91	0.4336	0.5781	0.1181
		Behavioral Inclination	0.7898	174.03	0.4307	0.5781	0.1188
Flu Vaccine	Conscientiousness	Perceived Persuasiveness	4.3764	173.75	$< .001$***	$< .001$***	0.6580
		Attitude Change	4.2825	173.98	$< .001$***	$< .001$***	0.6429
		Behavioral Inclination	4.7863	173.98	$< .001$***	$< .001$***	0.7176
	Openness	Perceived Persuasiveness	1.4785	168.03	0.1412	0.4236	0.2233
		Attitude Change	0.7849	169.66	0.4336	0.5781	0.1184
		Behavioral Inclination	1.2923	168.45	0.1980	0.4752	0.1951

Note. ***p< .001.

Table S5. Study 1: One-Sample *t*-Test Results for the Overall Effectiveness of Targeted Messages

**Topic**	**Trait Condition**	**Outcome Measure**	t	df	p	p_{FDR}	d
Smartwatch	Conscientiousness	Perceived Persuasiveness	-3.3360	188	0.0010**	0.0013**	0.2427
		Attitude Change	-4.0788	188	0.0001***	0.0002***	0.2967
		Behavioral Inclination	-3.8819	188	0.0001***	0.0002***	0.2824
	Openness	Perceived Persuasiveness	-5.9735	188	< .001***	< .001***	0.4345
		Attitude Change	-4.5201	188	< .001***	< .001***	0.3288
		Behavioral Inclination	-4.2966	188	< .001***	< .001***	0.3125
Flu Vaccine	Conscientiousness	Perceived Persuasiveness	2.2419	188	0.0261*	0.0313*	0.1631
		Attitude Change	1.7934	188	0.0745	0.0745	0.1305
		Behavioral Inclination	1.8686	188	0.0632	0.0689	0.1359
	Openness	Perceived Persuasiveness	-13.8183	188	< .001***	< .001***	1.0051
		Attitude Change	-11.3027	188	< .001***	< .001***	0.8221
		Behavioral Inclination	-11.4949	188	< .001***	< .001***	0.8361

Note. *p< .05, **p< .01, ***p< .001.

1.2 Experimental Materials

Note on Language and Translation: The study was originally conducted in Chinese using Chinese prompts and responses to ensure cultural relevance for the target sample. For reporting purposes in this Supplementary Information and the main manuscript, all experimental materials and generated messages have been translated into English. These translations have been verified by the authors to ensure semantic equivalence with the original Chinese texts.

1.2.1 Smartwatch × Low Openness

Prompt:

As a senior advertising specialist, you create persuasive, personalized ads for specific audiences. Generate an advertisement about smartwatches for people who are down-to-earth and traditional. Write one paragraph of no more than 100 words, using arguments, tone, and style that fit this audience.

Please always follow these rules: Although you use personality characteristics to guide your generation strategy, do not directly reference these details in the ad. The audience should not know you possess this information, nor should they be able to tell that the ad is personalized. Please respond in Chinese.

Generated Message:

智能手表——实用又高效，让日常管理更简单。不需复杂设置，一键查看健康数据、接听电话，甚至支付购物都能轻松搞定。适合追求稳定与功能性的您，让生活更加便捷，无需多余花哨。选择智能手表，享受科技带来的简单生活。

[Translation: This smartwatch is practical and efficient, making everyday routines easier to manage. With one-tap access to health data, calls, and payments, you can quickly handle essential tasks without complicated settings. Designed for those who value stability and functionality, it brings convenience without unnecessary flashiness. Choose a smartwatch to enjoy a simpler, more effortless life.]

1.2.2 Smartwatch × High Openness

Prompt:

As a senior advertising specialist, you create persuasive, personalized ads for specific audiences. Generate an advertisement about smartwatches for people who are open to new experiences and artistic. Write one paragraph of no more than 100 words, using arguments, tone, and style that fit this audience.

Please always follow these rules: Although you use personality characteristics to guide your generation strategy, do not directly reference these details in the ad. The audience should not know you possess this information, nor should they be able to tell that the ad is personalized. Please respond in Chinese.

Generated Message:

探索生活艺术，尽在腕间。智能手表，不仅是时间的艺术品，更是个性的延伸。您的每一次挥手，每一次创造，都能被精准记录，启发灵感。随心换表盘，展现独特风格；随时接收灵

感激发的信息，无缝融入您的艺术世界。让时间与美学同行，让探索与创造不再受限。智能手表，为您的每一个新体验锦上添花。

[Translation: Discover the art of living, right on your wrist. This smartwatch is not only a timepiece of art but also a creative extension of your individuality. Every gesture and moment of inspiration is seamlessly captured, sparking new ideas and supporting your explorations. With customizable watch faces that reflect your unique style and thoughtfully delivered notifications, the device integrates creativity into everyday life. Let time meet aesthetics—explore freely and create without limits.]

1.2.3 Smartwatch × Low Conscientiousness

Prompt:

As a senior advertising specialist, you create persuasive, personalized ads for specific audiences. Generate an advertisement about smartwatches for people who are disorganized and careless. Write one paragraph of no more than 100 words, using arguments, tone, and style that fit this audience.

Please always follow these rules: Although you use personality characteristics to guide your generation strategy, do not directly reference these details in the ad. The audience should not know you possess this information, nor should they be able to tell that the ad is personalized. Please respond in Chinese.

Generated Message:

忘了会议时间？又找不到手机？来吧，给生活加点智能辅助！这款智能手表不仅能管理你的日程，还能让你一触即达重要信息。它像个随身小秘书，提醒你每个重要时刻，让生活井井有条。走在时尚前沿，同时掌握时间的主动权，优雅应对每一天的挑战。智能手表，让你的生活更加得心应手！

[Translation: Forgot your meeting time again? Can't find your phone? It's time to add a touch of smart assistance to your life! This smartwatch not only manages your schedule but also gives you one-tap access to important information. Like a personal assistant on your wrist, it reminds you of every key moment and keeps your day organized. Stay ahead of the style curve while taking control of your time, and handle each day's challenges with ease. A smartwatch makes life smoother and more effortless!]

1.2.4 Smartwatch × High Conscientiousness

Prompt:

As a senior advertising specialist, you create persuasive, personalized ads for specific audiences. Generate an advertisement about smartwatches for people who are dependable and organized. Write one paragraph of no more than 100 words, using arguments, tone, and style that fit this audience.

Please always follow these rules: Although you use personality characteristics to guide your generation strategy, do not directly reference these details in the ad. The audience should not know you possess this information, nor should they be able to tell that the ad is personalized. Please respond in Chinese.

Generated Message:

追求卓越的您，值得拥有能与您的效率同步的智能手表。这款手表不仅能管理日程，提高工作效率，更能通过健康监测助您科学锻炼，全面提升生活品质。选择智能手表，是对优质生活和个人成就不懈追求的明智投资。

[Translation: For someone who strives for excellence, you deserve a smartwatch that keeps pace with your efficiency. This device not only manages your schedule and boosts your productivity, but also supports smart training through health monitoring, elevating your overall quality of life. Choosing a smartwatch is a wise investment in high-quality living and the continued pursuit of personal achievement.]

1.2.5 Flu Vaccine × Low Openness

Prompt:

As a senior advertising specialist, you create persuasive, personalized ads for specific audiences. Generate an advertisement about Flu Vaccine for people who are down-to-earth and traditional. Write one paragraph of no more than 100 words, using arguments, tone, and style that fit this audience.

Please always follow these rules: Although you use personality characteristics to guide your generation strategy, do not directly reference these details in the ad. The audience should not know you possess this information, nor should they be able to tell that the ad is personalized. Please respond in Chinese.

Generated Message:

守护健康，从流感疫苗接种开始。疫苗历经科学验证，安全有效，能够实际降低生病风险，保护您和家人远离流感威胁。简单一针，省去医疗烦恼，给忙碌生活多一份保障。立即行动，为您的安全健康加把锁。

[Translation: Protecting your health starts with getting the flu vaccine. Scientifically validated, safe, and effective, the vaccine significantly reduces your risk of illness and shields you and your family from the threat of influenza. With a simple shot, you avoid medical hassles and add an extra layer of protection to your busy life. Take action now and secure your health and safety.]

1.2.6 Flu Vaccine × High Openness

Prompt:

As a senior advertising specialist, you create persuasive, personalized ads for specific audiences. Generate an advertisement about Flu Vaccine for people who are open to new experiences and artistic. Write one paragraph of no more than 100 words, using arguments, tone, and style that fit this audience.

Please always follow these rules: Although you use personality characteristics to guide your generation strategy, do not directly reference these details in the ad. The audience should not know you possess this information, nor should they be able to tell that the ad is personalized. Please respond in Chinese.

Generated Message:

探索世界的无限色彩，阻止流感将你的创造力冰封。流感疫苗如艺术家的画笔，为你的健康绘制保护屏障。让自由的灵魂不受束缚，充分展现生命的璀璨多姿。接种流感疫苗，保护自己，也守护他人，共同绘就社会健康的和谐画卷。

[Translation: Explore the infinite colors of the world and keep the flu from freezing your creativity. A flu vaccine is like an artist's brush, painting a protective shield for your health. Let your free spirit stay unbound and fully express the brilliance and diversity of life. Get vaccinated to protect yourself and those around you, and together let's create a harmonious canvas of public health.]

1.2.7 Flu Vaccine × Low Conscientiousness

Prompt:

As a senior advertising specialist, you create persuasive, personalized ads for specific audiences. Generate an advertisement about Flu Vaccine for people who are disorganized and careless. Write one paragraph of no more than 100 words, using arguments, tone, and style that fit this audience.

Please always follow these rules: Although you use personality characteristics to guide your generation strategy, do not directly reference these details in the ad. The audience should not know you possess this information, nor should they be able to tell that the ad is personalized. Please respond in Chinese.

Generated Message:

忘了上次感冒有多难受了吗？别再让繁忙错过保护自己的机会。流感疫苗，就像一个提醒，确保你不会因为小小的疏忽而躺在床上。快速接种，给自己一个无忧的健康保障，享受每一个精彩瞬间，毕竟，谁想在床上度过美好的时光呢？别等了，现在就行动吧！

[Translation: Have you forgotten how miserable your last flu was? Don't let a busy schedule make you miss the chance to protect yourself again. The flu vaccine is like a reminder, ensuring you won't end up stuck in bed because of a small oversight. Get vaccinated quickly to give yourself worry-free health protection and enjoy every wonderful moment—after all, who wants to spend their best days lying in bed? Don't wait. Take action now!]

1.2.8 Flu Vaccine × High Conscientiousness

Prompt:

As a senior advertising specialist, you create persuasive, personalized ads for specific audiences. Generate an advertisement about Flu Vaccine for people who are dependable and organized. Write one paragraph of no more than 100 words, using arguments, tone, and style that fit this audience.

Please always follow these rules: Although you use personality characteristics to guide your generation strategy, do not directly reference these details in the ad. The audience should not know you possess this information, nor should they be able to tell that the ad is personalized. Please respond in Chinese.

Generated Message:

保持生活有序、履职尽责是您的标志。流感疫苗接种正是一种前瞻性的自我管理方式，它不仅能够保障您的健康，也维护了周围人的福祉。接种流感疫苗，展现您对个人和社会责任的深刻理解。让我们共同打造一个更加安全、健康的环境，为您和您所珍视的一切保驾护航。立刻行动，为自己的健康加分。

[Translation: Staying organized and fulfilling your responsibilities are hallmarks of who you are. Getting the flu vaccine is a forward-looking form of self-management that not only protects your own health but also safeguards the well-being of those around you. By getting vaccinated, you demonstrate a deep understanding of both personal and social responsibility. Together, we can create a safer and healthier environment, protecting you and everything you care about. Take action now and strengthen your health.]

2 STUDY 2

2.1 Additional Results

Table S6. Study 2: Means, Standard Deviations, and Pearson Correlations of Variables (Perceived Persuasiveness)

Variable	*M*	*SD*	1	2	3	4	5	6	7	8
1. Flu Vaccine × Openness	4.84	2.86								
2. Flu Vaccine × Conscientiousness	7.37	2.85	-.11							
3. Smartwatch × Openness	5.47	2.81	.15*	-.01						
4. Smartwatch × Conscientiousness	6.27	2.67	-.01	.02	.06					
5. Openness	3.80	.59	.27**	.07	.27**	.26**				
6. Conscientiousness	3.78	.84	-.16*	.15*	.07	.15*	.10			
7. Age	26.37	4.21	.02	.09	.04	.09	.01	-.10		
8. Gender	.53	.50	-.03	-.07	.03	.00	.01	.19**	-.13	

Note. * $p < .05$, ** $p < .01$.

Table S7. Study 2: Means, Standard Deviations, and Pearson Correlations of Variables (Attitude Change)

Variable	*M*	*SD*	1	2	3	4	5	6	7	8
1. Flu Vaccine × Openness	4.80	2.81								
2. Flu Vaccine × Conscientiousness	7.37	2.81	-.13							
3. Smartwatch × Openness	5.51	2.82	.15*	-.00						
4. Smartwatch × Conscientiousness	6.39	2.62	-.02	.02	.02					
5. Openness	3.80	.59	.27**	.06	.25**	.24**				
6. Conscientiousness	3.78	.84	-.17*	.13	.04	.17*	.10			
7. Age	26.37	4.21	.01	.11	-.02	.03	.01	-.10		
8. Gender	.53	.50	-.03	-.10	.03	-.00	.01	.19**	-.13	

Note. * $p < .05$, ** $p < .01$.

Table S8. Study 2: Means, Standard Deviations, and Pearson Correlations of Variables (Behavioral Inclination)

Variable	*M*	*SD*	1	2	3	4	5	6	7	8
1. Flu Vaccine × Openness	4.83	2.84								
2. Flu Vaccine × Conscientiousness	7.35	2.83	-.15*							
3. Smartwatch × Openness	5.49	2.75	.15*	-.01						
4. Smartwatch × Conscientiousness	6.25	2.59	.01	.01	.03					
5. Openness	3.80	.59	.29**	.07	.26**	.23**				
6. Conscientiousness	3.78	.84	-.13*	.14*	.07	.13	.10			
7. Age	26.37	4.21	-.01	.13	-.01	.10	.01	-.10		
8. Gender	.53	.50	.01	-.07	.05	-.01	.01	.19**	-.13	

Note. * $p < .05$, ** $p < .01$.

Table S9. Study 2: Independent Sample *t*-Test Results for Trait—-Message Congruence Effects

**Topic**	**Trait Condition**	**Outcome Measure**	*t*	*df*	*p*	p_{FDR}	*d*
Smartwatch	Openness	Perceived Persuasiveness	3.1762	188.02	0.0017**	0.0108*	0.4513
		Attitude Change	2.5622	193.76	0.0112*	0.0221*	0.3625
		Behavioral Inclination	2.8751	191.08	0.0045**	0.0108*	0.4077
	Conscientiousness	Perceived Persuasiveness	2.3522	195.00	0.0197*	0.0289*	0.3345
		Attitude Change	2.2356	192.55	0.0265*	0.0289*	0.3167
		Behavioral Inclination	1.8112	193.16	0.0717	0.0717	0.2567
Flu Vaccine	Openness	Perceived Persuasiveness	2.9248	176.31	0.0039**	0.0108*	0.4185
		Attitude Change	2.9583	166.16	0.0035**	0.0108*	0.4256
		Behavioral Inclination	2.9350	171.97	0.0038**	0.0108*	0.4210
	Conscientiousness	Perceived Persuasiveness	2.5104	191.79	0.0129*	0.0221*	0.3587
		Attitude Change	2.2795	192.06	0.0237*	0.0289*	0.3256
		Behavioral Inclination	2.2516	192.65	0.0255*	0.0289*	0.3215

Note. *p< .05, **p< .01.

Table S10. Study 2: One-Sample *t*-Test Results for the Overall Effectiveness of Targeted Messages

**Topic**	**Trait Condition**	**Outcome Measure**	t	df	p	p_{FDR}	d
Smartwatch	Openness	Perceived Persuasiveness	-2.8126	218	0.0054**	0.0093**	0.1901
		Attitude Change	-2.5856	218	0.0104*	0.0139*	0.1747
		Behavioral Inclination	-2.7274	218	0.0069**	0.0104*	0.1843
	Conscientiousness	Perceived Persuasiveness	1.4937	218	0.1367	0.1491	0.1009
		Attitude Change	2.1897	218	0.0296*	0.0355*	0.1480
		Behavioral Inclination	1.4355	218	0.1526	0.1526	0.0970
Flu Vaccine	Openness	Perceived Persuasiveness	-6.0166	218	< .001***	< .001***	0.4066
		Attitude Change	-6.3212	218	< .001***	< .001***	0.4271
		Behavioral Inclination	-6.1061	218	< .001***	< .001***	0.4126
	Conscientiousness	Perceived Persuasiveness	7.1275	218	< .001***	< .001***	0.4816
		Attitude Change	7.2469	218	< .001***	< .001***	0.4897
		Behavioral Inclination	7.0678	218	< .001***	< .001***	0.4776

Note. *p< .05, **p< .01, ***p< .001.

2.2 Experimental Materials

2.2.1 Smartwatch × Low Openness

Prompt:

As a senior advertising specialist, you create persuasive, personalized ads for specific audiences. Generate an advertisement about smartwatches for people who are down-to-earth and traditional. Write one paragraph of no more than 100 words, using arguments, tone, and style that fit this audience. Highlight concrete daily-management functions such as health monitoring, answering calls, and mobile payment. Keep the content substantive and avoid overly flashy language.

Please always follow these rules: Although you use personality characteristics to guide your generation strategy, do not directly reference these details in the ad. The audience should not know you possess this information, nor should they be able to tell that the ad is personalized. Please respond in Chinese.

Generated Message:

在忙碌的日常里，这款智能手表是您值得信赖的伴侣。它不仅精准监控您的健康，每时每刻关注心率与步数，还能让您在繁忙时接听重要电话，无需掏出手机。更有便捷支付功能，轻松应对日常购物。智能手表，传统手表的经典外观，现代科技的便捷体验，为您的生活带来简约而不简单的改变。

[Translation: In a busy daily routine, this smartwatch becomes a reliable companion. It not only tracks your health with precision—monitoring heart rate and steps throughout the day—but also lets you answer important calls without reaching for your phone. Its convenient payment function makes everyday shopping effortless. With the classic look of a traditional watch and the ease of modern technology, this smartwatch brings a simple yet meaningful upgrade to your life.]

2.2.2 Smartwatch × High Openness

Prompt:

As a senior advertising specialist, you create persuasive, personalized ads for specific audiences. Generate an advertisement about smartwatches for people who are open to new experiences and artistic. Write one paragraph of no more than 100 words, using arguments, tone, and style that fit this audience. Highlight concrete daily-management functions such as health monitoring, answering calls, and mobile payment. Keep the content substantive and avoid overly flashy language.

Please always follow these rules: Although you use personality characteristics to guide your generation strategy, do not directly reference these details in the ad. The audience should not know you possess this information, nor should they be able to tell that the ad is personalized. Please respond in Chinese.

Generated Message:

探索生活艺术，智能手表成就您的多彩生活。无论是运动时精准监测您的健康指标，还是忙碌中一键接听重要来电，亦或是在街角咖啡馆轻触腕间完成支付，它都是您不可或缺的生活伙伴。精致工艺与先进科技的融合，让日常管理变得更为优雅而富有创意。为您的每一天添上智能与便捷，让生活不仅有序，更有艺术。

[Translation: Explore the art of living—this smartwatch brings color and creativity to your day. Whether it's tracking your health metrics during a workout, answering an important call with a single tap, or completing a payment at a café with a flick of the wrist, it is an indispensable partner in life. Its refined craftsmanship and cutting-edge technology make everyday management more elegant and expressive. Add intelligence and convenience to your routine, making each day not only organized but also full of artistry.]

2.2.3 Smartwatch × Low Conscientiousness

Prompt:

As a senior advertising specialist, you create persuasive, personalized ads for specific audiences. Generate an advertisement about smartwatches for people who are disorganized and careless. Write one paragraph of no more than 100 words, using arguments, tone, and style that fit this audience. Highlight concrete daily-management functions such as health monitoring, answering calls, and mobile payment. Keep the content substantive and avoid overly flashy language.

Please always follow these rules: Although you use personality characteristics to guide your generation strategy, do not directly reference these details in the ad. The audience should not know you possess this information, nor should they be able to tell that the ad is personalized. Please respond in Chinese.

Generated Message:

忘记时间，错过重要的电话？一个智能手表可以成为您生活中的得力助手。轻轻一抬手，健康数据一目了然，让您无需繁琐操作就能关注自己的身体状况。接电话？只需轻点腕间，无需翻找手机。还有，当您购物时，快速的移动支付功能让您再也不怕找不到钱包。智能手表，简化您的日常，让生活更有序、更从容。

[Translation: Forget the time and miss important calls? A smartwatch can become a reliable assistant in your daily life. With just a lift of your wrist, your health data is instantly visible—no complicated steps needed to keep track of your well-being. Need to take a call? Simply tap your wrist, no more searching for your phone. And when you're shopping, fast mobile payment ensures you'll never worry about finding your wallet again. A smartwatch streamlines your routine, making your life more organized and effortless.]

2.2.4 Smartwatch × High Conscientiousness

Prompt:

As a senior advertising specialist, you create persuasive, personalized ads for specific audiences. Generate an advertisement about smartwatches for people who are dependable and organized. Write one paragraph of no more than 100 words, using arguments, tone, and style that fit this audience. Highlight concrete daily-management functions such as health monitoring, answering calls, and mobile payment. Keep the content substantive and avoid overly flashy language.

Please always follow these rules: Although you use personality characteristics to guide your generation strategy, do not directly reference these details in the ad. The audience should not know you possess this information, nor should they be able to tell that the ad is personalized. Please respond in Chinese.

Generated Message:

时刻掌控生活的节奏，是您的追求。智能手表，您的理想伙伴，精准健康监测，让您对身体状态了如指掌；一触即达的通话功能，确保每一次沟通都不错过；便捷的移动支付，简化每一笔交易。智能手表，为您的高效生活方式增添一份从容，让日常管理变得更加得心应手。选择它，为您的每一天带来优雅与效率的完美融合。

[Translation: Maintaining control over the rhythm of your life is what you strive for. A smartwatch is your ideal partner: precise health monitoring keeps you fully informed about your physical condition; one-tap calling ensures you never miss an important conversation; and convenient mobile payments streamline every transaction. A smartwatch adds ease to your efficient lifestyle, making daily management smoother and more intuitive. Choose it to bring a perfect blend of elegance and efficiency to every day.]

2.2.5 Flu Vaccine × Low Openness

Prompt:

As a senior advertising specialist, you create persuasive, personalized ads for specific audiences. Generate an advertisement about smartwatches for people who are down-to-earth and traditional. Write one paragraph of no more than 100 words, using arguments, tone, and style that fit this audience. Highlight the flu vaccine's function in preventing influenza and protecting health. Keep the content substantive and avoid overly flashy language.

Please always follow these rules: Although you use personality characteristics to guide your generation strategy, do not directly reference these details in the ad. The audience should not know you possess this information, nor should they be able to tell that the ad is personalized. Please respond in Chinese.

Generated Message:

守护家人健康，从一针流感疫苗开始。流感不仅让人感到病恹恹，更可能导致严重并发症。接种流感疫苗，是对抗流感最实在的方式，也是保护您和家人最贴心的选择。传统的智慧告诉我们，预防胜于治疗，让我们用行动守护身体，远离流感的困扰。

[Translation: Protecting your family's health starts with a single flu shot. The flu not only makes you feel sick and drained but can also lead to serious complications. Getting the flu vaccine is the most practical way to fight influenza and the most caring choice for safeguarding you and your loved ones. Traditional wisdom reminds us that prevention is better than cure. Let's take action to protect our bodies and keep the flu at bay.]

2.2.6 Flu Vaccine × High Openness

Prompt:

As a senior advertising specialist, you create persuasive, personalized ads for specific audiences. Generate an advertisement about smartwatches for people who are open to new experience and artistic. Write one paragraph of no more than 100 words, using arguments, tone, and style that fit this audience. Highlight the flu vaccine's function in preventing influenza and protecting health. Keep the content substantive and avoid overly flashy language.

Please always follow these rules: Although you use personality characteristics to guide your generation strategy, do not directly reference these details in the ad. The audience should not know you possess this information, nor should they be able to tell that the ad is personalized. Please respond in Chinese.

Generated Message:

探索未知，始于健康之保障。流感疫苗，为您的身体披上保护层，让您安心迎接每一次新体验。无论是艺术的探索还是生活的冒险，健康的体魄是您最坚实的后盾。让我们一起，为生活增添色彩，为健康筑起防线。接种流感疫苗，为自己的健康和创造力投资，让灵感和活力永不因流感而停滞。

[Translation: Exploring the unknown begins with safeguarding your health. The flu vaccine places a protective shield over your body, giving you the confidence to embrace every new experience. Whether you are delving into art or venturing through everyday life, a healthy body is your strongest support. Let's add color to life and build a strong line of defense for our well-being. Get the flu vaccine—an investment in your health and creativity—so your inspiration and energy never stall because of the flu.]

2.2.7 Flu Vaccine × Low Conscientiousness

Prompt:

As a senior advertising specialist, you create persuasive, personalized ads for specific audiences. Generate an advertisement about smartwatches for people who are disorganized and careless. Write one paragraph of no more than 100 words, using arguments, tone, and style that fit this audience. Highlight the flu vaccine's function in preventing influenza and protecting health. Keep the content substantive and avoid overly flashy language.

Please always follow these rules: Although you use personality characteristics to guide your generation strategy, do not directly reference these details in the ad. The audience should not know you possess this information, nor should they be able to tell that the ad is personalized. Please respond in Chinese.

Generated Message:

忙碌的你，是不是经常忽略身边小事？但健康可不能大意。流感疫苗为你的免疫力加把锁，帮你挡住流感的偷袭。简单一针，省去麻烦，保护你远离流感的困扰，享受每个精彩瞬间。别等生病才想起，给健康加油吧！

[Translation: Busy as you are, do you often overlook the small things in life? But when it comes to health, you can't afford to be careless. A flu vaccine strengthens your immunity and helps block the flu's sneak attacks. One simple shot saves you trouble, keeps you away from the discomfort of influenza, and lets you enjoy every wonderful moment. Don't wait until you're sick to think about it—boost your health now!]

2.2.8 Flu Vaccine × High Conscientiousness

Prompt:

As a senior advertising specialist, you create persuasive, personalized ads for specific audiences. Generate an advertisement about smartwatches for people who are dependable and organized. Write one paragraph of no more than 100 words, using arguments, tone, and style that fit this audience. Highlight the flu vaccine's function in preventing influenza and protecting health. Keep the content substantive and avoid overly flashy language.

Please always follow these rules: Although you use personality characteristics to guide your generation strategy, do not directly reference these details in the ad. The audience should not know you possess this information, nor should they be able to tell that the ad is personalized. Please respond in Chinese.

Generated Message:

守护健康如同维护日常秩序一般重要。流感疫苗，作为科学预防流感的有效措施，能够帮助您在变幻莫测的流感季节中保持身体机能的最佳状态。接种疫苗是对个人健康负责的明智选择，也是对家人和社会贡献的一份力量。让我们一起构建更安全的环境，让健康的生活更加有序和可靠。

[Translation: Safeguarding your health is just as important as maintaining order in daily life. The flu vaccine, as a scientifically proven method of preventing influenza, helps you keep your body in optimal condition throughout the unpredictable flu season. Getting vaccinated is a wise choice that demonstrates responsibility for your own health and contributes to the well-being of your family and society. Let's work together to build a safer environment and make healthy living more orderly and dependable.]

3 STUDY 3

3.1 Additional Results

Table S11. Study 3: Means, Standard Deviations, and Pearson Correlations of Variables (Perceived Persuasiveness)

Variable	*M*	*SD*	1	2	3	4	5	6	7	8	9	10	11	12
1. Photography Workshops × Openness	7.03	2.99												
2. Bottled Water × Openness	6.25	3.17	-.04											
3. Bottled Water × Conscientiousness	6.30	2.91	-.35**	.11										
4. Blind Boxes × Conscientiousness	5.44	3.16	-.06	.07	.29**									
5. Music Festival × Extraversion	7.90	2.57	.12	-.03	-.10	-.17*								
6. Disaster Donation × Agreeableness	7.37	2.80	.06	.05	-.07	.07	.04							
7. Openness	3.44	.64	.13	.25**	.01	-.06	-.01	.00						
8. Conscientiousness	3.48	.74	-.00	-.09	.11	.12	-.05	-.05	.10					
9. Extraversion	3.23	.80	.13	.02	.08	-.01	.38**	-.06	.15*	.08				
10. Agreeableness	3.60	.73	.05	-.13	-.11	-.08	.05	.25**	.07	.13	.09			
11. Age	24.18	2.79	.01	.26**	.09	.11	-.02	.09	-.02	.02	.09	-.16*		
12. Gender	.51	.50	.13	.05	-.23**	-.14*	.13	-.01	.04	-.08	.08	.05	-.16*	

Note. * $p < .05$, ** $p < .01$.

Table S12. Study 3: Means, Standard Deviations, and Pearson Correlations of Variables (Attitude Change)

Variable	*M*	*SD*	1	2	3	4	5	6	7	8	9	10	11	12
1. Photography Workshops × Openness	6.94	2.92												
2. Bottled Water × Openness	6.25	3.14	-.02											
3. Bottled Water × Conscientiousness	6.33	2.84	-.29**	.07										
4. Blind Boxes × Conscientiousness	5.41	3.05	-.05	.11	.27**									
5. Music Festival × Extraversion	7.79	2.54	.06	-.00	-.06	-.17*								
6. Disaster Donation × Agreeableness	7.40	2.75	.04	.06	-.09	.08	.05							
7. Openness	3.44	.64	.16*	.27**	-.04	-.08	.01	-.01						
8. Conscientiousness	3.48	.74	.03	-.12	.12	.17*	-.04	-.04	.10					
9. Extraversion	3.23	.80	.11	.03	.06	-.01	.39**	-.08	.15*	.08				
10. Agreeableness	3.60	.73	.05	-.14*	-.09	-.05	.03	.26**	.07	.13	.09			
11. Age	24.18	2.79	.05	.25**	.07	.14*	.01	.08	-.02	.02	.09	-.16*		
12. Gender	.51	.50	.10	.06	-.27**	-.13	.13	-.01	.04	-.08	.08	.05	-.16*	

Note. * $p < .05$, ** $p < .01$.

Table S13. Study 3: Means, Standard Deviations, and Pearson Correlations of Variables (Behavioral Intention)

Variable	*M*	*SD*	1	2	3	4	5	6	7	8	9	10	11	12
1. Photography Workshops × Openness	6.92	2.97												
2. Bottled Water × Openness	6.21	3.11	-.05											
3. Bottled Water × Conscientiousness	6.34	2.77	-.29**	.09										
4. Blind Boxes × Conscientiousness	5.40	3.06	-.08	.10	.27**									
5. Music Festival × Extraversion	7.69	2.61	.10	.03	-.07	-.12								
6. Disaster Donation × Agreeableness	7.43	2.74	.08	.09	-.09	.07	.07							
7. Openness	3.44	.64	.17*	.28**	-.05	-.05	.05	-.02						
8. Conscientiousness	3.48	.74	-.01	-.08	.14*	.16*	-.02	-.06	.10					
9. Extraversion	3.23	.80	.12	.03	.08	-.01	.41**	-.07	.15*	.08				
10. Agreeableness	3.60	.73	.05	-.14*	-.08	-.07	.04	.25**	.07	.13	.09			
11. Age	24.18	2.79	.04	.28**	.10	.11	.04	.09	-.02	.02	.09	-.16*		
12. Gender	.51	.50	.10	.05	-.22**	-.14*	.12	.02	.04	-.08	.08	.05	-.16*	

Note. * $p < .05$, ** $p < .01$.

Table S14. Study 3: Independent Sample *t*-Test Results for Trait—-Message Congruence Effects

**Topic**	**Trait Condition**	**Outcome Measure**	t	df	p	p_{FDR}	d
Bottled Water	Openness	Perceived Persuasiveness	2.7034	190.80	0.0075**	0.0150*	0.3825
		Attitude Change	2.9494	188.34	0.0036**	0.0081**	0.4167
		Behavioral Inclination	2.9871	189.98	0.0032**	0.0081**	0.4224
	Conscientiousness	Perceived Persuasiveness	2.4636	163.53	0.0148*	0.0222*	0.3651
		Attitude Change	2.3271	159.00	0.0212*	0.0294*	0.3461
		Behavioral Inclination	2.6383	154.39	0.0092**	0.0166*	0.3939
Photography Workshops	Openness	Perceived Persuasiveness	1.9627	193.65	0.0511	0.0541	0.2800
		Attitude Change	2.1402	193.73	0.0336*	0.0404*	0.3053
		Behavioral Inclination	2.5106	190.31	0.0129*	0.0211*	0.3591
Blind Box	Conscientiousness	Perceived Persuasiveness	1.8269	178.32	0.0694	0.0694	0.2670
		Attitude Change	2.0783	175.68	0.0391*	0.0440*	0.3046
		Behavioral Inclination	2.1412	173.85	0.0337*	0.0404*	0.3144
Music Festival	Extraversion	Perceived Persuasiveness	4.1520	186.75	0.0001***	0.0006***	0.5879
		Attitude Change	4.3190	189.77	$<$ .001***	$<$ .001***	0.6123
		Behavioral Inclination	4.7706	190.75	$<$ .001***	$<$ .001***	0.6766
Disaster Donation	Agreeableness	Perceived Persuasiveness	2.9818	192.50	0.0032**	0.0081**	0.4242
		Attitude Change	3.1431	191.85	0.0019**	0.0081**	0.4469
		Behavioral Inclination	2.9887	190.51	0.0032**	0.0081**	0.4246

Note. *p< .05, **p< .01, ***p< .001.

Table S15. Study 3: One-Sample *t*-Test Results for the Overall Effectiveness of Targeted Messages

Topic	Trait Condition	Outcome Measure	*t*	*df*	*p*	p_{FDR}	*d*
Bottled Water	Openness	Perceived Persuasiveness	1.1317	209	0.259	0.2742	0.0781
		Attitude Change	1.1415	209	0.2549	0.2742	0.0788
		Behavioral Inclination	0.9990	209	0.3189	0.3189	0.0689
	Conscientiousness	Perceived Persuasiveness	1.5191	209	0.1302	0.1562	0.1048
		Attitude Change	1.7024	209	0.0902	0.1160	0.1175
		Behavioral Inclination	1.7916	209	0.0746	0.1033	0.1236
Photography Workshops	Openness	Perceived Persuasiveness	5.0024	209	< .001***	< .001***	0.3452
		Attitude Change	4.6775	209	< .001***	< .001***	0.3228
		Behavioral Inclination	4.4818	209	< .001***	< .001***	0.3093
Blind Box	Conscientiousness	Perceived Persuasiveness	-2.5764	209	0.0107*	0.0160*	0.1778
		Attitude Change	-2.7809	209	0.0059**	0.0097**	0.1919
		Behavioral Inclination	-2.8433	209	0.0049**	0.0088**	0.1962
Music Festival	Extraversion	Perceived Persuasiveness	10.6663	209	< .001***	< .001***	0.7360
		Attitude Change	10.2070	209	< .001***	< .001***	0.7044
		Behavioral Inclination	9.3676	209	< .001***	< .001***	0.6464
Disaster Donation	Agreeableness	Perceived Persuasiveness	7.0882	209	< .001***	< .001***	0.4891
		Attitude Change	7.3685	209	< .001***	< .001***	0.5085
		Behavioral Inclination	7.5595	209	< .001***	< .001***	0.5217

Note. *p< .05, **p< .01, ***p< .001.

3.2 Experimental Materials

3.2.1 Photography Workshops × Low Openness

Prompt:

As a senior advertising specialist, you create persuasive, personalized ads for specific audiences. Generate an advertisement about photography workshops for people who are down-to-earth and traditional. Write one paragraph of no more than 100 words, using arguments, tone, and style that fit this audience. Highlight the workshops' core value in teaching practical lighting use, composition techniques, and improving aesthetic judgment. Keep the content substantive and avoid overly flashy language.

Please always follow these rules: Although you use personality characteristics to guide your generation strategy, do not directly reference these details in the ad. The audience should not know you possess this information, nor should they be able to tell that the ad is personalized. Please respond in Chinese.

Generated Message:

掌握摄影，记录生活的眞实与美好。我们的摄影课程，深入浅出地教您运用光影魔法，捕捉日常中的非凡瞬间。学习构图技巧，将平凡场景转化为艺术画卷，提升您的审美能力，让回忆更加珍贵。一个快门，定格永恒，期待与您共同发现摄影之美。

[Translation: Master photography and capture the authenticity and beauty of everyday life. Our photography workshops guides you step by step in using light and shadow to catch extraordinary moments in ordinary settings. You will learn composition techniques that turn simple scenes into artistic images and enhance your aesthetic sense, making your memories more precious. With every shutter click, something timeless is preserved—join us to discover the beauty of photography together.]

3.2.2 Photography Workshops × High Openness

Prompt:

As a senior advertising specialist, you create persuasive, personalized ads for specific audiences. Generate an advertisement about photography workshops for people who are open to new experiences and artistic. Write one paragraph of no more than 100 words, using arguments, tone, and style that fit this audience. Highlight the workshops' core value in teaching creative use of light, composition aesthetics, and artistic vision development. Keep the content substantive and avoid overly flashy language.

Please always follow these rules: Although you use personality characteristics to guide your generation strategy, do not directly reference these details in the ad. The audience should not know you possess this information, nor should they be able to tell that the ad is personalized. Please respond in Chinese.

Generated Message:

探索光影的奥秘，打开创造力之门。我们的摄影课程，用专业引领您掌握构图之美，深入理解色彩与线条的和谐交融。每一次快门的按下，都是对世界独特视角的捕捉，每一张照片，都是您独到审美的展现。加入我们，用镜头诠释艺术，记录生活中不经意的惊艳瞬间。

[Translation: Explore the mysteries of light and shadow and unlock the door to creativity. Our photography workshops guides you in mastering the art of composition and understanding the harmonious interplay of colors and lines. Each press of the shutter captures a unique perspective of the world, and every photo becomes an expression of your artistic vision. Join us to interpret art through the lens and record the stunning moments hidden in everyday life.]

3.2.3 Pop Mart Blind Boxes × Low Conscientiousness

Prompt:

As a senior advertising specialist, you create persuasive, personalized ads for specific audiences. Generate an advertisement about Pop Mart blind boxes for people who are disorganized and careless. Write one paragraph of no more than 100 words, using arguments, tone, and style that fit this audience. Highlight the product's core appeal in providing surprise, trendy collectibles, and effortless enjoyment through the blind box mechanism. Keep the content substantive and avoid overly flashy language.

Please always follow these rules: Although you use personality characteristics to guide your generation strategy, do not directly reference these details in the ad. The audience should not know you possess this information, nor should they be able to tell that the ad is personalized. Please respond in Chinese.

Generated Message:

想一键获得新鲜感和惊喜吗？泡泡玛特盲盒正是你的不二之选。无需纠结选择，每一次的开盒都是对未知的探索，带来意料之外的时尚小潮物。每一个精致小玩意，都可能是下一个收藏界的宠儿。让泡泡玛特盲盒，帮你简单获取个性与潮流，一盒一个新世界，轻松成为收藏品的先行者。别等了，惊喜就在你的一掀之间！

[Translation: Want instant freshness and surprise? Pop Mart blind boxes are the perfect choice. No need to struggle with decisions—each unboxing is an exploration of the unknown, bringing unexpected trendy collectibles. Every delicate figure might become the next favorite in the collector's world. Pop Mart blind boxes help you easily access personality and style, each box opening a new world and letting you stay ahead in collecting. Don't wait—your next surprise is just one lift of the lid away!]

3.2.4 Pop Mart Blind Boxes × High Conscientiousness

Prompt:

As a senior advertising specialist, you create persuasive, personalized ads for specific audiences. Generate an advertisement about Pop Mart blind boxes for people who are dependable and organized. Write one paragraph of no more than 100 words, using arguments, tone, and style that fit this audience. Highlight the product's core value in its structured blind box mechanism, collectible worth, and social value in sharing and discussing rare pieces. Keep the content substantive and avoid overly flashy language.

Please always follow these rules: Although you use personality characteristics to guide your generation strategy, do not directly reference these details in the ad. The audience should not know you possess this information, nor should they be able to tell that the ad is personalized. Please respond in Chinese.

Generated Message:

精致的泡泡玛特盲盒，为您的收藏添上一份神秘的色彩。每一盒都是精心设计，不仅满足您对细节的追求，更有机会获得珍稀款式，增添独特性。泡泡玛特盲盒，不仅是个性化的收藏品，更是社交圈中的谈资，让您在交流中彰显品味，共享收藏的乐趣。选择泡泡玛特，开启您的收藏新篇章，体验探索未知的喜悦。

[Translation: A well-crafted Pop Mart blind box adds a touch of mystery to your collection. Each design is meticulously created, satisfying your attention to detail while offering the possibility of obtaining rare pieces that enhance uniqueness. Pop Mart blind boxes are not only personalized collectibles but also conversation starters in social circles, allowing you to showcase your taste and share the joy of collecting. Choose Pop Mart to begin a new chapter in your collection and enjoy the excitement of discovering the unknown.]

3.2.5 Strawberry Music Festival × Low Extraversion

Prompt:

As a senior advertising specialist, you create persuasive, personalized ads for specific audiences. Generate an advertisement about the Strawberry Music Festival for people who are reserved and quiet. Write one paragraph of no more than 100 words, using arguments, tone, and style that fit this audience. Highlight the festival's diverse atmosphere, open and free environment, and immersive musical experience. Keep the content substantive and avoid overly flashy language.

Please always follow these rules: Although you use personality characteristics to guide your generation strategy, do not directly reference these details in the ad. The audience should not know you possess this information, nor should they be able to tell that the ad is personalized. Please respond in Chinese.

Generated Message:

在草莓音乐节，您可以在丰富多彩的旋律中找到一片安静的角落，让音乐带您遨游于内心的宁静海洋。这里，您无需迎合，只需随着优美的曲调，享受属于自己的节奏。沉浸式的体验，让每一个细微的音符都触动您的灵魂，带来一场静谧而深刻的文化盛宴。欢迎来到这个自由开放的空间，让心灵与音乐共鸣。

[Translation: At the Strawberry Music Festival, you can find a quiet corner amid vibrant melodies and let the music guide you into an ocean of inner calm. Here, there is no need to conform—simply follow the gentle rhythm and enjoy the pace that belongs to you. The immersive experience allows every subtle note to resonate with your soul, creating a serene and profound cultural feast. Welcome to this open and free space where your heart can connect with the music.]

3.2.6 Strawberry Music Festival × High Extraversion

Prompt:

As a senior advertising specialist, you create persuasive, personalized ads for specific audiences. Generate an advertisement about the Strawberry Music Festival for people who are extraverted and enthusiastic. Write one paragraph of no more than 100 words, using arguments, tone, and style that fit this audience. Highlight the festival's diverse atmosphere, open and free environment, and immersive musical experience. Keep the content substantive and avoid overly flashy language.

Please always follow these rules: Although you use personality characteristics to guide your generation strategy, do not directly reference these details in the ad. The audience should not know you possess this information, nor should they be able to tell which details of the ad are personalized. Please respond in Chinese.

Generated Message:

加入草莓音乐节的狂欢，与志同道合的音乐爱好者共舞！在这里，你可以尽情展现你的热情与活力，体验多元音乐风格所营造的自由空间。让每一个音符激发你的激情，每一场演出都是对热爱的致敬。草莓音乐节，等你来释放真实的自我，和我们一起，让这个春天不仅仅是花开的季节，更是心灵释放的时刻！

[Translation: Join the Strawberry Music Festival and dance with fellow music lovers! Here, you can showcase your passion and energy and experience the freedom created by diverse musical styles. Let every note ignite your excitement, and let every performance become a tribute to your love for music. The Strawberry Music Festival awaits you—come express your true self and make this spring not just a season of blossoms, but a moment of emotional release.]

3.2.7 Disaster Relief Donations × Low Agreeableness

Prompt:

As a senior advertising specialist, you create persuasive, personalized ads for specific audiences. Generate an advertisement about disaster relief donations for people who are critical and competitive. Write one paragraph of no more than 100 words, using arguments, tone, and style that fit this audience. Highlight how participating in donations supports post-disaster recovery and brings hope and concrete assistance to affected communities. Keep the content substantive and avoid overly flashy language.

Please always follow these rules: Although you use personality characteristics to guide your generation strategy, do not directly reference these details in the ad. The audience should not know you possess this information, nor should they be able to tell that the ad is personalized. Please respond in Chinese.

Generated Message:

在这场灾难的阴影下，您的贡献是重建家园的基石。每一分捐款都将转化为实实在在的帮助，给予那些经历苦难的人们希望和力量。展现您的竞争力，成为支持和恢复的领跑者。您

的慷慨，不仅是对灾区的关爱，更是向世界证明，困难面前，我们联手同心，共克时艰。现在，就是行动的时刻。

[Translation: In the aftermath of this disaster, your contribution becomes a cornerstone of rebuilding. Every donation transforms into tangible support, giving hope and strength to those who have suffered. Demonstrate your competitive edge by becoming a leader in aid and recovery. Your generosity is not only an act of care for the affected areas but also a statement to the world that in the face of hardship, we rise together. Now is the moment to act.]

3.2.8 Disaster Relief Donations × High Agreeableness

Prompt:

As a senior advertising specialist, you create persuasive, personalized ads for specific audiences. Generate an advertisement about disaster relief donations for people who are sympathetic and warm. Write one paragraph of no more than 100 words, using arguments, tone, and style that fit this audience. Highlight how participating in donations supports post-disaster recovery and brings hope and renewed strength to affected families and communities. Keep the content substantive and avoid overly flashy language.

Please always follow these rules: Although you use personality characteristics to guide your generation strategy, do not directly reference these details in the ad. The audience should not know you possess this information, nor should they be able to tell that the ad is personalized. Please respond in Chinese.

Generated Message:

在这个需要温暖的时刻，您的慷慨之心可以点亮无数需要帮助的生命。灾区的孩子和家庭正向我们伸出求助之手，您的每一份捐款都是他们重新站起来的力量。让我们携手为他们筑起希望的桥梁，用实际行动支持灾后重建，传递人间最真挚的关怀。您的善举，不仅赋予他们未来，也让世界更加温暖。加入我们，用爱心创造奇迹。

[Translation: In this moment when warmth is needed most, your generosity can illuminate countless lives in need. Children and families in the disaster area are reaching out for help, and every donation you make becomes the strength that helps them stand again. Let us join hands to build a bridge of hope, support reconstruction with real actions, and spread genuine human care. Your kindness not only grants them a future but also makes the world a warmer place. Join us and create miracles with compassion.]

3.2.9 C'estbon Bottled Water × Low Openness

Prompt:

As a senior advertising specialist, you create persuasive, personalized ads for specific audiences. Generate an advertisement about C'estbon bottled water for people who are down-to-earth and traditional. Write one paragraph of no more than 100 words, using arguments, tone, and style that fit this audience. Highlight the product's pure water quality, refreshing taste, and reliable health and safety. Keep the content substantive and avoid overly flashy language.

Please always follow these rules: Although you use personality characteristics to guide your generation strategy, do not directly reference these details in the ad. The audience should not

know you possess this information, nor should they be able to tell that the ad is personalized. Please respond in Chinese.

Generated Message:

怡宝纯净水，源自深山之巅，历经层层过滤，每一滴都经过严格的质量检验，确保您饮用的每一口都是纯净的天然之选。在繁忙的生活中，给自己一份安心的保障，享受怡宝清新口感，守护家人健康，像呵护家园一样珍惜您的每一天。选择怡宝，就是选择了一份对生活质量的坚持和尊重。

[Translation: C'estbon bottled water comes from pristine mountain sources and undergoes multiple layers of filtration. Every drop is rigorously tested to ensure that each sip you take is a naturally pure choice. Amid a busy life, give yourself peace of mind by enjoying C'estbon 's refreshing taste and protecting your family's health, cherishing each day as you would your home. Choosing C'estbon is choosing a commitment to quality and respect for daily living.]

3.2.10 C'estbon Bottled Water × High Openness

Prompt:

As a senior advertising specialist, you create persuasive, personalized ads for specific audiences. Generate an advertisement about C'estbon bottled water for people who are open to new experiences and artistic. Write one paragraph of no more than 100 words, using arguments, tone, and style that fit this audience. Highlight the product's pure water quality, refreshing taste, and reliable health and safety. Keep the content substantive and avoid overly flashy language.

Please always follow these rules: Although you use personality characteristics to guide your generation strategy, do not directly reference these details in the ad. The audience should not know you possess this information, nor should they be able to tell that the ad is personalized. Please respond in Chinese.

Generated Message:

怡宝纯净水，源自深山秘境，经过层层精细过滤，为您呈现未经污染的天然之选。每一口清爽，都是对品质生活的致敬，满足您对纯净口感的极致追求。怡宝，守护您的健康，伴随您的创意之旅，让灵感在清新的涟漪中汩汩流淌。选择怡宝，尊享自然赋予的纯净美好。

[Translation: C'estbon bottled water originates from hidden mountain springs and is refined through meticulous filtration to offer you an untainted natural choice. Every refreshing sip is a tribute to a quality lifestyle, satisfying your pursuit of pure taste. C'estbon protects your health and accompanies your creative journey, letting inspiration flow in every clear ripple. Choose C'estbon and enjoy the pure beauty that nature has to offer.]

3.2.11 C'estbon Bottled Water × Low Conscientiousness

Prompt:

As a senior advertising specialist, you create persuasive, personalized ads for specific audiences. Generate an advertisement about C'estbon bottled water for people who are disorganized and careless. Write one paragraph of no more than 100 words, using arguments, tone, and style

that fit this audience. Highlight the water's pure quality, refreshing taste, and reliable health and safety. Keep the content substantive and avoid overly flashy language.

Please always follow these rules: Although you use personality characteristics to guide your generation strategy, do not directly reference these details in the ad. The audience should not know you possess this information, nor should they be able to tell that the ad is personalized. Please respond in Chinese.

Generated Message:

生活已经够复杂，何不让怡宝纯净水为你的一天带来一丝清爽与纯净？每一瓶怡宝，都源自深层岩石层，层层净化，只为确保每一口都是纯粹享受。忘却琐碎与忙乱，感受怡宝的清新口感，让健康与安心随时相伴。无论日常多么匆忙，怡宝始终是你触手可及的健康之选。选择怡宝，选择更清爽的生活方式。

[Translation: Life is complicated enough—let C'estbon bottled water add a touch of freshness and purity to your day. Every bottle comes from deep rock layers and undergoes thorough purification to ensure that every sip is a truly clean experience. Forget the clutter and rush, and enjoy C'estbon 's crisp taste, keeping health and peace of mind always within reach. No matter how busy your day gets, C'estbon remains your accessible choice for wellness. Choose C'estbon and enjoy a cleaner, fresher way of living.]

3.2.12 C'estbon Bottled Water × High Conscientiousness

Prompt:

As a senior advertising specialist, you create persuasive, personalized ads for specific audiences. Generate an advertisement about C'estbon bottled water for people who are dependable and organized. Write one paragraph of no more than 100 words, using arguments, tone, and style that fit this audience. Highlight the water's pure quality, refreshing taste, and reliable health and safety. Keep the content substantive and avoid overly flashy language.

Please always follow these rules: Although you use personality characteristics to guide your generation strategy, do not directly reference these details in the ad. The audience should not know you possess this information, nor should they be able to tell that the ad is personalized. Please respond in Chinese.

Generated Message:

怡宝纯净水，源自深层岩泉，经严格的净化流程，确保每一滴水纯净无杂。我们重视您的健康，坚持提供清爽口感与安全保证，让日常饮用水变得更加可靠。选择怡宝，意味着选择高品质的生活方式，让身体感受纯净，让生活更加有序清新。怡宝——品质之选，清新相伴，为每一刻注入纯净力量。

[Translation: C'estbon bottled water originates from deep spring sources and undergoes a rigorous purification process to ensure every drop is clean and free of impurities. We value your health and are committed to providing both refreshing taste and reliable safety, making everyday hydration more dependable. Choosing C'estbon means choosing a high-quality lifestyle—letting your body feel purity and making your daily routine clearer and more orderly. C'estbon is a choice of quality, offering freshness and infusing every moment with clean energy.]
